# Objective Measures of Physical Functioning, Disabilities in Daily Life and Trends During Ageing: A Repeated Cross‐Sectional Study

**DOI:** 10.1002/jcsm.70133

**Published:** 2025-11-23

**Authors:** Liangyu Yin, Jinghong Zhao

**Affiliations:** ^1^ Department of Nephrology, Chongqing Key Laboratory of Prevention and Treatment of Kidney Disease, Chongqing Clinical Research Center of Kidney and Urology Diseases Xinqiao Hospital, Army Medical University (Third Military Medical University) Chongqing China

**Keywords:** ageing, activities of daily living, chair stand test, functional capacity, gait speed, physical function

## Abstract

**Background:**

Maintenance of adequate physical functioning is key for lifelong health, particularly for an ageing society with increasing health care needs. We aimed to assess the association of two objective physical function (PF) measures with functional capacity (FC) outcomes, while also exploring the underlying trends.

**Methods:**

This was a repeated cross‐sectional study. We used data from a nationally representative survey of middle‐aged and older Chinese individuals across 150 counties or districts across 28 provinces of China. Longitudinal data from three waves collected in 2011 (baseline, *n* = 4753), 2013 (*n* = 4922) and 2015 (*n* = 6165) were used for parallel analysis. PF was measured using the five‐time chair stand test (CST) and gait speed (GS). FC outcomes were assessed using 20 items related to activities of daily living or instrumental activities of daily living. We conducted various analyses, including dose‐dependent analyses, cutoff development, multivariate analyses and causal inference, to systematically examine the associations across multiple dimensions.

**Results:**

A total of 15 840 participants from three waves were analysed. FC limitations increased as the study population aged. The most frequently reported limitation across all three waves was the inability to jog 1 km (59.2%, 60.3% and 60.7%, respectively). Compared to low GS, prolonged CST time was more strongly associated with a greater number of FC limitation items (2011, 20 vs. 16; 2013, 20 vs. 16; 2015, 20 vs. 20). The associations between both PF measures and outcomes were predominantly nonlinear and dose‐dependent (all *p* < 0.05). Overall, CST showed superior performance relative to GS for diagnosing different FC limitations. The optimal global cutoffs were 0.66 m/s for GS and 10.85 s for CST. Controlling for age, sex and BMI, we noted that CST had generally higher standardized odds ratios than GS in predicting FC outcomes. Causal inference analysis revealed that CST had a stronger direct causal effect on overall FC than GS, both at the global and local levels in all three waves (for instance, average treatment effect in the baseline survey, CST = 0.729, 95% CI = 0.470–0.989, GS = 0.235, 95% CI = 0.033–0.438).

**Conclusions:**

PF measures have varied performance to reflect the diversity and magnitude of activities individuals actually perform. CST has stronger associations with multidimensional FC than GS despite the ageing process. This superiority also exists in those activities that do not primarily involve the lower limb musculoskeletal system. These findings are crucial for health stakeholders to develop more targeted strategies for managing functional limitations.

## 1 Introduction

The capacity to maintain adequate physical functioning and mobility independence is crucial for lifelong health [1, 2, 3]. Ageing is a leading cause of functional limitations, which are linked to an increased risk of disability, cognitive impairment, institutionalization and early mortality [4, 5, 6]. Alarmingly, the global ageing population is projected to reach 1.5 billion by 2050 [7]. Addressing the unique needs of this growing demographic and promoting functional health to facilitate healthy ageing have become significant global public health challenges for policymakers [8]. Assessing physical function (PF) is also crucial in general research and clinical settings. PF is a significant predictor of clinical outcomes [9]. Randomized controlled trials frequently use PF as a main endpoint [10]. Notably, PF has been incorporated into patient management protocols [10, 11, 12, 13], popular clinical guidelines [14, 15, 16, 17, 18] and international consensus statements [19, 20, 21] for diagnosing conditions such as malnutrition [17, 18], frailty [14, 15, 16] and sarcopenia [19, 20, 21, 22].

Although PF can be measured subjectively (e.g., questionnaires) [17, 18, 23, 24], these methods are prone to biases that often overestimate the intensity and duration of physical activity [23, 24]. Recent recommendations have emphasized the use of objective PF tests [2, 25, 26], such as usual gait speed (GS), the 6‐min walk test, the stair‐climb power test, the timed‐up‐and‐go test, the five‐time chair stand test (CST) and the Short Physical Performance Battery [2, 5, 19, 20, 21, 26]. Among these, GS and CST are widely used due to their reliability, speed, cost‐effectiveness and simplicity [9, 27]. Although some studies and international guidelines treat GS and CST as interchangeable PF measures [9, 20, 21], their comparative superiority remains unclear [19, 20, 21]. A key shared feature of GS and CST is their reliance on the lower limb musculoskeletal system during execution. However, health is a complex, multidimensional process and PF‐related health outcomes do not occur in isolation [28]. In some cases, a single PF measure may fail to capture the full complexity of functional capacity (FC). Thus, linking objectively measured PF with real‐world, multidimensional FC outcomes is crucial to determine whether measures in controlled environments truly reflect the diversity and magnitude of activities individuals perform in daily life [29]. Activities of daily living (ADL) and instrumental activities of daily living (IADL) are multidimensional indicators of FC and have been widely used as outcome measures of functional limitations [2, 22, 26]. They also reflect dependency, care burden and morbidity compression in populations, which are critical concerns for public health [30]. However, the limitation of prior research lies in analysing aggregated ADL/IADL scores [2, 26, 29, 30], which fail to provide deeper insights into the quantified relationship between PF and each distinct FC item.

To address these gaps, this study systematically identified, quantified and compared associations of GS and CST with health outcomes represented by 20 FC items. The study aimed to: 1) identify the superior PF measure and its optimal cutoff for clinical relevance, 2) identify FC outcomes most strongly associated with PF; and 3) estimate temporal trends in PF–FC associations among Chinese adults between 2011 and 2015. These findings are key for strategically informing the prevention, diagnosis, surveillance and intervention of functional limitations in various settings.

## 2 Methods

### 2.1 Study Design and Participants

This was an observational, multicenter study using data from the baseline and follow‐up surveys of the China Health and Retirement Longitudinal Study (CHARLS) completed in 2011, 2013 and 2015. CHARLS is an ongoing, nationally representative longitudinal survey in China focused on ageing‐related policy and health issues [31]. In 2011, CHARLS recruited participants from 10 257 households located in 150 counties or districts and 450 villages or urban communities across 28 provinces using multistage stratified probability‐proportionate‐to‐size sampling in China. Through structured questionnaires and in‐person interviews, high‐quality data were collected from a nationally representative sample of Chinese adults aged 45 years and older, covering sociodemographic, lifestyle and health‐related information. A total of 17 708 individuals were successfully interviewed during the 2011 baseline survey. Follow‐up surveys were conducted every 2 years after the baseline survey, following the same ascertainment and assessment protocols. Participants were interviewed in their usual residence by interviewers trained by CHARLS staff members at Peking University, Beijing, China. Full details of the study design, methods and response rates have been previously described [32].

**FIGURE 1:**
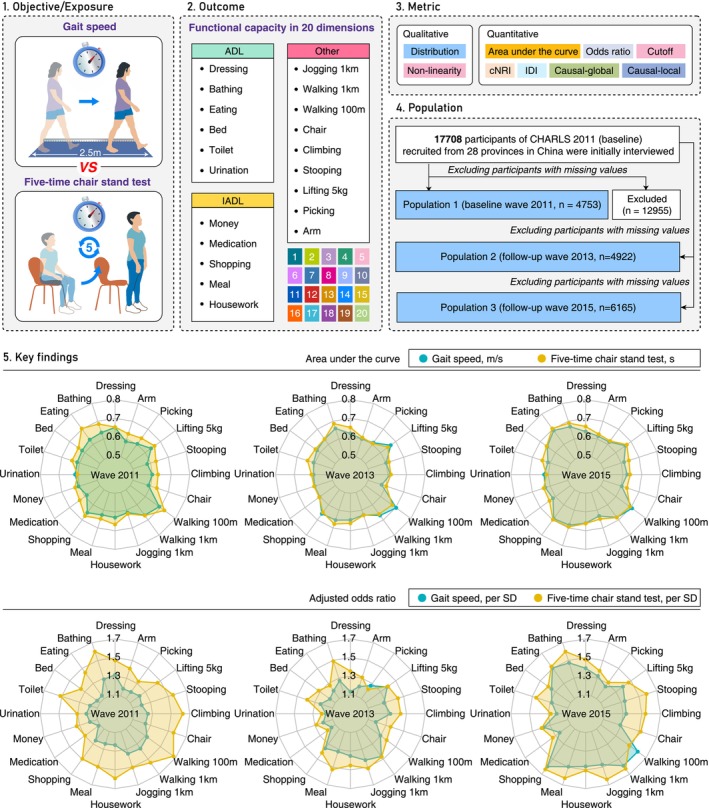
Graphical abstract of the study. ADL, activities of daily living; IADL, instrumental activities of daily living; cNRI, continuous net reclassification improvement; IDI, integrated discrimination improvement; CHARLS, the China Health and Retirement Longitudinal Study; SD, standard deviation.

In this study, participants with missing data on study variables were excluded. A graphical abstract illustrating subject inclusion is provided in Figure 1. The research protocol of CHARLS received approval from the Biomedical Ethics Committee of Peking University (Approval Number: IRB00001052‐11015). All participants or their legal representatives signed written informed consent forms to participate in the baseline and follow‐up surveys. This study followed the Strengthening the Reporting of Observational Studies in Epidemiology (STROBE) guidelines.

### 2.2 Data Handling

Age (years), sex, body height, body weight, GS, CST and 20 FC items were extracted from the CHARLS database. Body weight and height were measured using a stadiometer and a digital floor scale, respectively, to the nearest 0.1 cm and 0.1 kg. Body mass index (BMI) was calculated as the weight in kilograms divided by the height in meters squared. BMI (kg/m^2^) was also categorized as underweight (< 18.5), normal (18.5 to < 24), overweight (24 to < 28) or obese (≥28) according to Chinese recommendations [33].

GS and CST were measured following standard protocols under the guidance of interviewers trained by the CHARLS team [32]. The respondent was instructed to walk 2.5 m along a 4 m, noncarpeted walking course for two trials at their usual GS. GS (m/s) was calculated as 2.5 m divided by the average time of the first and second trials in seconds. CST indicates the number of seconds it took the respondent to complete five consecutive chair stands. The respondent was instructed to keep their arms folded across their chest, stand up straight and then sit down again at their fastest pace five times without stopping in between and without using their arms to push off. GS and CST were analysed as continuous and binary variables. The cutoffs for dichotomizing GS and CST were based on the Asian Working Group for Sarcopenia 2019 framework [20]. The optimal cutoffs of GS and CST were also explored using the receiver operating characteristic (ROC) curve by maximizing the Youden index. A cutoff for each of the 20 FC items was derived independently; then the mean of these 20 cutoff values across the three waves was calculated to determine the global threshold for subsequent analysis.

The 20 FC items in CHARLS include 6 ADLs (dressing, bathing, eating, bed, toilet and urination), 5 IADLs (money, medication, shopping, meal and housework) and 9 other items (jogging 1 km, walking 1 km, walking 100 m, chair, climbing, stooping, lifting 5 kg, picking and arm). These items include tasks such as getting in and out of bed, managing money and standing up from a chair after sitting for long periods. Respondents who reported any difficulty in completing these tasks were recorded as positive (1 point); otherwise, they were recorded as negative (0 point). A summary FC score was defined as the summed points of all 20 FC items. Detailed descriptions of the 20 FC items are provided in Table S1.

### 2.3 Statistical Analysis

All statistical analyses were performed in parallel for the baseline and two follow‐up surveys. Continuous data are presented as medians (interquartile range, IQR) and were compared using the nonparametric Wilcoxon's rank‐sum test. Categorical data were expressed as numbers (percentages) and compared using the chi‐squared test. A restricted cubic spline (RCS, with four knots at the 5th, 35th, 65th and 95th percentiles) was used to flexibly analyse the potential nonlinear associations of GS and CST with the 20 FC items. The potential nonlinearity was tested using a likelihood ratio test, with *p* < 0.05 indicating a nonlinear relationship. ROC curves were utilized to determine the performance for GS and CST in predicting the 20 FC items. Area under the curve (AUC) and 95% confidence interval (CI) were calculated to quantify the discrimination performance. Differences between correlated ROC curves were assessed using Delong's test. Integrated discrimination improvement (IDI) and continuous net reclassification improvement (cNRI) were used to statistically compare the discriminatory performance. AUC calculations were based on 1000 iterations of bootstrap resampling, while IDI and cNRI were adjusted with 1000 iterations of perturbation‐resampling to obtain unbiased estimates.

The associations between GS/CST and FC were evaluated using multivariable logistic regressions. Odds ratios (OR) with 95% CI were calculated to estimate the effects. Two types of models were developed with different numbers of covariates: Model 1 (unadjusted crude model) and Model 2 (adjusted for age at baseline, sex and BMI). To make the results comparable, GS and CST were analysed as per standard deviation in the logistic regression models. The causal effect of GS/CST on FC was analysed by estimating the conditional average treatment effect (ATE) at the global level and treatment effect at the local level using a double machine learning (DML) causal inference framework. DML offers an advantage in robust causal inference by effectively controlling for high‐dimensional covariates and confounders, while avoiding overfitting. A detailed description of the causal effect modelling is shown in Table S2. All reported *P* values were two‐sided and considered significant at *p* < 0.05. All statistical analyses were performed using R (version 4.3.1, Foundation for Statistical Computing, Vienna, Austria) or Python (version 3.9.11, The Python Software Foundation, USA).

## 3 Results

### 3.1 Population Overview

A total of 15 840 participants across three waves were included in our analysis (2011 wave = 4753, 2013 wave = 4922 and 2015 wave = 6165, Figure 1). In the 2011 wave, 53% (2517/4753) of participants were men and 47% (2236/4753) of participants were women, with a total median age of 65.0 years (IQR: 62.0–71.0). A total of 56% (2660/4753) participants had a normal BMI. The medians (IQRs) for GS and CST were 0.6 (0.5, 0.8) (m/s) and 10.6 (8.8, 13.3) (s), respectively. Among the 20 FC items, jogging 1 km had the highest positivity rate (59.2%, 2816/4753), while the fewest participants reported difficulty with eating (1.9%, 92/4753) (Table 1). The distributions of the above variables were generally similar across the three waves. As ageing progresses, there is a trend of increasing positivity rates for these 20 FC indicators. For instance, the positivity rate for walking 1 km increased from 11.3% in 2011 to 17.6% in 2013 and then to 20.9% in 2015 (Tables S3–S4).

**TABLE 1:** Association of objectively measured physical function with different factors in the 2011 wave.

		Gait speed (normal: ≥1 m/s)			Five‐time chair stand test (normal: <12 s)		
Characteristics	Overall (*n* = 4753)	Normal (*n* = 258)	Impaired (*n* = 4495)	*P*	Normal (*n* = 3049)	Impaired (*n* = 1704)	*P*
Age, years	65.0 [62.0, 71.0]	64.0 [61.0, 68.0]	65.0 [62.0, 71.0]	<0.001	64.0 [61.0, 69.0]	67.0 [63.0, 73.0]	<0.001
Sex, men	2517 (53.0)	184 (71.3)	2333 (51.9)	<0.001	1770 (58.1)	747 (43.8)	<0.001
Body mass index, kg/m^2^	22.4 [20.1, 25.0]	22.9 [20.6, 25.2]	22.4 [20.1, 25.0]	0.059	22.5 [20.3, 25.1]	22.2 [19.9, 24.9]	0.053
Body mass index category				0.032			0.028
I underweight	481 (10.1)	16 (6.2)	465 (10.3)		288 (9.4)	193 (11.3)	
II normal	2660 (56.0)	140 (54.3)	2520 (56.1)		1707 (56.0)	953 (55.9)	
III overweight	1208 (25.4)	82 (31.8)	1126 (25.1)		807 (26.5)	401 (23.5)	
IV obese	404 (8.5)	20 (7.8)	384 (8.5)		247 (8.1)	157 (9.2)	
Body height, m	1.6 [1.5, 1.6]	1.6 [1.6, 1.7]	1.6 [1.5, 1.6]	<0.001	1.6 [1.5, 1.6]	1.5 [1.5, 1.6]	<0.001
Body weight, kg	55.1 [48.4, 63.3]	59.5 [52.0, 67.1]	54.9 [48.2, 62.9]	<0.001	55.7 [49.1, 63.7]	53.9 [46.9, 62.1]	<0.001
Gait speed, m/s	0.6 [0.5, 0.8]	1.1 [1.0, 1.2]	0.6 [0.5, 0.7]	<0.001	1.5 [1.2, 1.8]	1.8 [1.5, 2.3]	<0.001
Five‐time chair stand test, s	10.6 [8.8, 13.3]	8.4 [7.0, 10.2]	10.7 [8.9, 13.4]	<0.001	9.3 [7.9, 10.5]	14.3 [13.1, 16.6]	<0.001
ADL							
Dressing	188 (4.0)	1 (0.4)	187 (4.2)	0.004	81 (2.7)	107 (6.3)	<0.001
Bathing	227 (4.8)	5 (1.9)	222 (4.9)	0.041	87 (2.9)	140 (8.2)	<0.001
Eating	92 (1.9)	1 (0.4)	91 (2.0)	0.104	29 (1.0)	63 (3.7)	<0.001
Bed	196 (4.1)	3 (1.2)	193 (4.3)	0.022	91 (3.0)	105 (6.2)	<0.001
Toilet	589 (12.4)	12 (4.7)	577 (12.8)	<0.001	282 (9.2)	307 (18.0)	<0.001
Urination	216 (4.5)	9 (3.5)	207 (4.6)	0.494	117 (3.8)	99 (5.8)	0.002
IADL							
Money	619 (13.0)	12 (4.7)	607 (13.5)	<0.001	294 (9.6)	325 (19.1)	<0.001
Medication	307 (6.5)	7 (2.7)	300 (6.7)	0.017	141 (4.6)	166 (9.7)	<0.001
Shopping	368 (7.7)	5 (1.9)	363 (8.1)	0.001	149 (4.9)	219 (12.9)	<0.001
Meal	339 (7.1)	10 (3.9)	329 (7.3)	0.049	148 (4.9)	191 (11.2)	<0.001
Housework	371 (7.8)	5 (1.9)	366 (8.1)	<0.001	157 (5.1)	214 (12.6)	<0.001
Other							
Jogging 1 km	2816 (59.2)	104 (40.3)	2712 (60.3)	<0.001	1623 (53.2)	1193 (70.0)	<0.001
Walking 1 km	536 (11.3)	7 (2.7)	529 (11.8)	<0.001	232 (7.6)	304 (17.8)	<0.001
Walking 100 m	99 (2.1)	1 (0.4)	98 (2.2)	0.082	27 (0.9)	72 (4.2)	<0.001
Chair	1303 (27.4)	36 (14.0)	1267 (28.2)	<0.001	671 (22.0)	632 (37.1)	<0.001
Climbing	2092 (44.0)	55 (21.3)	2037 (45.3)	<0.001	1135 (37.2)	957 (56.2)	<0.001
Stooping	1482 (31.2)	45 (17.4)	1437 (32.0)	<0.001	770 (25.3)	712 (41.8)	<0.001
Lifting 5 kg	536 (11.3)	9 (3.5)	527 (11.7)	<0.001	242 (7.9)	294 (17.3)	<0.001
Picking	163 (3.4)	5 (1.9)	158 (3.5)	0.239	76 (2.5)	87 (5.1)	<0.001
Arm	461 (9.7)	14 (5.4)	447 (9.9)	0.023	225 (7.4)	236 (13.8)	<0.001

Abbreviations: ADL, activities of daily living; IADL, instrumental activities of daily living; Other, other functional capacity items.

### 3.2 Physical Function and Functional Capacity

Lower GS and longer CST times were associated with impaired FC across all three waves (Figure S1). Specifically, lower GS was linked to an increased positivity in 16 FC items in 2011 (except for eating, urination, walking 100 m and picking), 16 items in 2013 (except for dressing, eating, bed and picking) and 20 items in 2015 (all *p* < 0.05). In contrast, longer CST times were associated with increased positivity in all 20 FC items in 2011, 2013 and 2015 (all *p* < 0.05) (Table 1, Tables S3–S4).

### 3.3 Nonlinear Associations

**FIGURE 2:**
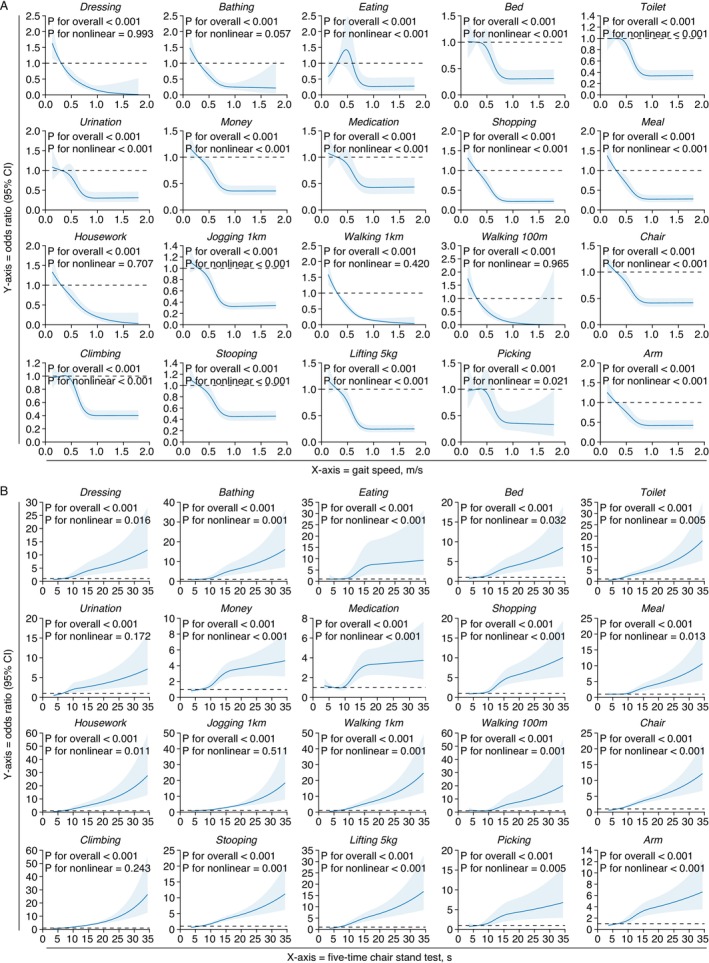
Restricted cubic spine analysis on the association of gait speed and chair stand test with 20 functional capacity outcomes in the 2011 wave. (A) Gait speed and outcomes. (B) Chair stand test and outcomes. RCS, restricted cubic spine.

Potential nonlinear associations of GS and CST with the 20 FC items across the three waves were analyzed independently (Figure 2, Figure S2–S3). In the 2011 wave, RCS analysis revealed that lower GS values were associated with increased odds of impaired FC (all *P* overall < 0.05), with either linear (*n* = 5, *P* nonlinear > 0.05) or nonlinear (*n* = 15, *P* nonlinear < 0.05) relationships. Similar results were observed for the 2013 wave (all *P* overall < 0.05, 8 linear and 12 nonlinear) and the 2015 wave (all *P* overall < 0.05, 13 linear and 7 nonlinear). In contrast, longer CST times were associated with increased odds of impaired FC (all *P* overall < 0.05, 3 linear and 17 nonlinear) in the 2011 wave. Similar results were observed for the 2013 wave (20 nonlinear, all *P* overall < 0.05) and the 2015 wave (20 nonlinear, all *P* overall < 0.05).

### 3.4 Diagnostic Performance

The performance of GS and CST in diagnosing FC impairment was compared. GS demonstrated the highest diagnostic performance for the walking 100 m item in the 2011 (AUC = 0.692, 95% CI = 0.641–0.742), 2013 (AUC = 0.703, 95% CI = 0.669–0.737) and 2015 waves (AUC = 0.707, 95% CI = 0.682–0.732). In contrast, CST showed superior performance for the walking 100 m item in the 2011 wave (AUC = 0.726, 95% CI = 0.672–0.780), for bathing in the 2013 wave (AUC = 0.686, 95% CI = 0.653–0.718) and for eating in the 2015 wave (AUC = 0.704, 95% CI = 0.661–0.747) (Table 2, Table S5–S6).

**TABLE 2:** Diagnostic performance of objective physical function measures on multidimensional functional capacity in the 2011 wave.

	Gait speed, m/s		Five‐time chair stand test, s		Between‐model comparison (reference = gait speed)				
Index	AUC (95% CI)	Cutoff	AUC (95% CI)	Cutoff	*P*‐AUC	cNRI (95% CI)	*P*‐cNRI	IDI (95% CI)	*P*‐IDI
ADL									
Dressing	0.652 (0.613–0.691)	0.59	0.654 (0.614–0.695)	12.47	0.912	0.174 (0.028–0.320)	0.019	0.006 (−0.003–0.015)	0.201
Bathing	0.636 (0.600–0.672)	0.51	0.684 (0.647–0.721)	11.08	0.011	0.374 (0.242–0.507)	<0.001	0.019 (0.008–0.029)	<0.001
Eating	0.631 (0.578–0.683)	0.57	0.704 (0.655–0.752)	11.02	0.006	0.548 (0.347–0.749)	<0.001	0.005 (0.002–0.009)	<0.001
Bed	0.618 (0.579–0.657)	0.50	0.642 (0.603–0.682)	11.00	0.244	0.255 (0.113–0.398)	<0.001	0.011 (0.005–0.016)	<0.001
Toilet	0.613 (0.590–0.636)	0.66	0.642 (0.619–0.665)	10.41	0.030	0.259 (0.173–0.345)	<0.001	0.027 (0.020–0.034)	<0.001
Urination	0.614 (0.576–0.653)	0.57	0.601 (0.564–0.639)	9.82	0.549	0.134 (−0.003–0.270)	0.055	0.005 (−0.001–0.012)	0.105
IADL									
Money	0.606 (0.583–0.629)	0.63	0.625 (0.602–0.649)	10.94	0.148	0.233 (0.149–0.317)	<0.001	0.011 (0.006–0.015)	<0.001
Medication	0.587 (0.555–0.619)	0.68	0.634 (0.601–0.667)	13.66	0.009	0.374 (0.260–0.489)	<0.001	0.007 (0.004–0.009)	<0.001
Shopping	0.652 (0.624–0.681)	0.51	0.674 (0.645–0.703)	10.94	0.181	0.359 (0.254–0.465)	<0.001	0.019 (0.011–0.026)	<0.001
Meal	0.627 (0.596–0.658)	0.55	0.649 (0.617–0.680)	11.07	0.194	0.289 (0.179–0.399)	<0.001	0.019 (0.012–0.027)	<0.001
Housework	0.630 (0.602–0.659)	0.55	0.667 (0.638–0.696)	11.02	0.018	0.350 (0.244–0.455)	<0.001	0.028 (0.019–0.037)	<0.001
Other									
Jogging 1 km	0.614 (0.598–0.631)	0.68	0.621 (0.605–0.637)	9.82	0.472	0.126 (0.068–0.184)	<0.001	0.017 (0.011–0.023)	<0.001
Walking 1 km	0.669 (0.645–0.693)	0.52	0.671 (0.647–0.695)	10.96	0.902	0.171 (0.081–0.261)	<0.001	0.020 (0.011–0.029)	<0.001
Walking 100 m	0.692 (0.641–0.742)	0.54	0.726 (0.672–0.780)	11.99	0.223	0.425 (0.228–0.623)	<0.001	0.024 (0.006–0.041)	0.007
Chair	0.588 (0.570–0.606)	0.59	0.635 (0.617–0.652)	10.41	<0.001	0.295 (0.232–0.358)	<0.001	0.030 (0.024–0.036)	<0.001
Climbing	0.599 (0.583–0.615)	0.62	0.630 (0.614–0.646)	11.26	<0.001	0.262 (0.206–0.319)	<0.001	0.038 (0.031–0.044)	<0.001
Stooping	0.581 (0.564–0.598)	0.66	0.630 (0.613–0.647)	11.50	<0.001	0.269 (0.208–0.329)	<0.001	0.033 (0.027–0.039)	<0.001
Lifting 5 kg	0.638 (0.614–0.663)	0.60	0.661 (0.638–0.685)	10.41	0.083	0.273 (0.184–0.363)	<0.001	0.024 (0.016–0.031)	<0.001
Picking	0.603 (0.560–0.646)	0.63	0.640 (0.596–0.685)	13.49	0.111	0.261 (0.106–0.417)	0.001	0.003 (−0.002–0.007)	0.199
Arm	0.586 (0.559–0.614)	0.61	0.629 (0.603–0.656)	10.85	0.002	0.232 (0.136–0.327)	0.001	0.013 (0.009–0.018)	0.001

Abbreviations: ADL, activities of daily living; IADL, instrumental activities of daily living; Other, other functional capacity items.

Overall, CST outperformed GS in diagnostic accuracy based on AUC metrics across all waves (Figure 1). In the 2011 wave, DeLong's test indicated that CST outperformed GS for 9 of the 20 FC indicators (all *p* < 0.05), while performance was comparable for the remaining 11 (all *p* > 0.05). cNRI and IDI analyses further supported CST's superior diagnostic performance (cNRI: 18 superior, 2 comparable; IDI: 17 superior, 3 comparable) (Table 2). Similar trends were observed in the 2013 (DeLong's test: 2 superior, 18 comparable; cNRI: 14 superior, 6 comparable; IDI: 10 superior, 10 comparable) and 2015 waves (DeLong's test: 5 superior, 15 comparable; cNRI: 11 superior, 8 comparable, 1 inferior; IDI: 8 superior, 11 comparable, 1 inferior) (Tables S5–S6).

### 3.5 Cutoff Optimization

**FIGURE 3:**
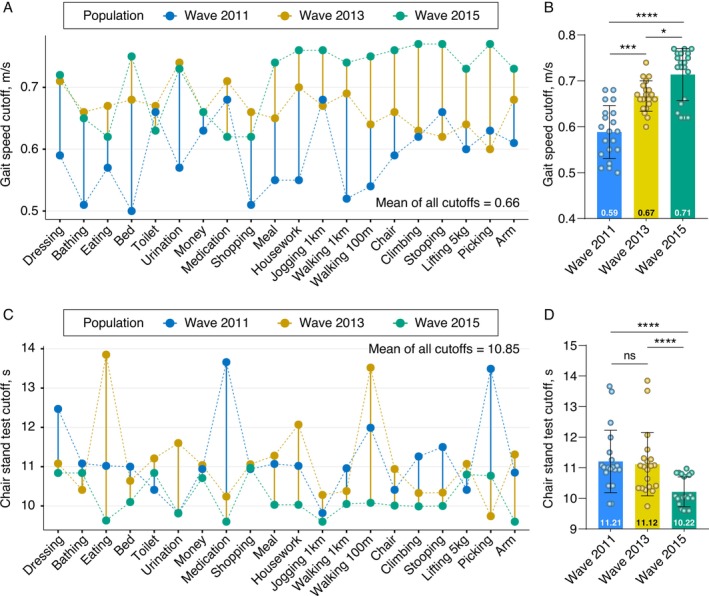
Cutoffs and trend analysis. (A) Cutoffs of gait speed for the 20 functional capacity outcomes across the three waves. (B) Variation in the mean cutoff of gait speed across the three waves. (C) Cutoffs of the chair stand test for the 20 functional capacity outcomes across the three waves. (D) Variation in the mean cutoff of the chair stand test across the three waves.

The optimal cutoffs for GS and CST in diagnosing impaired FC were calculated for each of the 20 FC items across the three waves (Table 2, Table S5–S6, Figure 3). The median of the 20 optimal cutoff values for GS was 0.59 m/s (range: 0.50–0.68), 0.67 m/s (range: 0.60–0.71) and 0.71 m/s (range: 0.62–0.77) for the three waves, respectively. The mean of all 60 cutoff values from the three waves was 0.66 m/s. For CST, the median of the 20 optimal cutoff values was 11.21 s (range: 9.82–13.66), 11.12 s (range: 9.74–13.85) and 10.22 s (range: 9.60–10.97) for the three waves, respectively. The mean of all 60 cutoff values from the three waves was 10.85 s.

Impaired GS (<0.66 m/s) and CST (≥10.85 s) were then redefined using the calculated optimal cutoff values, and their associations with various factors were analyzed. Consistent results were observed for both GS and CST, showing that impaired PF was associated with all 20 FC items across all three waves (all *p* < 0.05) (Table S7–S9).

### 3.6 Multivariable Analysis

**FIGURE 4:**
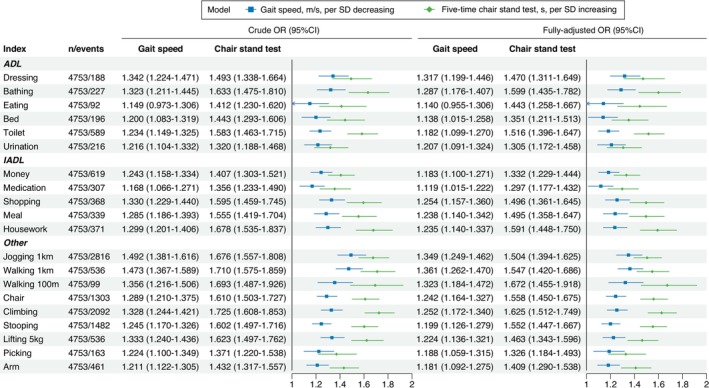
Univariate and multivariate logistic regression analysis on the association of standardized gait speed and chair stand test with 20 function capacity outcomes in the 2011 wave. ADL, activities of daily living; IADL, instrumental activities of daily living; SD, standard deviation.

Multivariable logistic regression analyses were performed to examine the associations of standardized GS (per SD decrease) and CST (per SD increase) with the 20 FC items across the three waves. GS and CST were positively associated with the positivity of all FC items across the three waves, except for the GS and eating pair in the 2011 wave (Figure 4, Figure S4–S5). For point estimation of effect size, CST showed higher odds ratios (ORs) than GS across all 20 FC items in the 2011 wave. This superiority of CST persisted across 19 items in both the 2013 (except the picking item) and 2015 (except the walking 100 m item) waves (Figure 1).

### 3.7 Causal Relationship

In the 2011 wave, causal analysis revealed that CST had a higher ATE (0.729, 95% CI = 0.470–0.989) compared to GS (0.235, 95% CI = 0.033–0.438) on overall FC at the global level (Figure S6A). Further analysis of a randomly selected participant showed similar results at the individual level (treatment effect: CST = 0.669, 95% CI = 0.406–0.932; GS = 0.205, 95% CI = −0.060–0.469) (Figure S6B). Subsequent analyses of the 2013 (ATE: CST = 0.571, 95% CI = 0.392–0.750; GS = 0.396, 95% CI = 0.224–0.569; treatment effect: CST = 0.595, 95% CI = 0.434–0.756; GS = 0.388, 95% CI = 0.215–0.560) (Figure S6C–S6D) and 2015 (ATE: CST = 0.851, 95% CI = 0.601–1.101; GS = 0.575, 95% CI = 0.375–0.774; treatment effect: CST = 0.939, 95% CI = 0.719–1.159; GS = 0.472, 95% CI = 0.353–0.591) waves yielded consistent results (Figure S6E–S6F).

## 4 Discussion

This was a multicenter study using nationally representative data collected under a standardized protocol. We investigated the associations of two objective PF measures with 20 clinical outcomes, using multiple metrics across three longitudinal waves of participants. Over 1000 relationships were systematically analysed and compared. These results collectively suggest that CST is generally a better objective PF measure than GS despite the ageing process. Additionally, using nationally representative samples, we calculated cutoff values for CST and GS at different levels. We also provided standardization in PF which helps unravel dose–response relationships and inform guidelines [26]. Altogether, the results provide compelling evidence that could deepen the understanding of disease and health frameworks that include PF as a component. Importantly, outcomes represented by ADL and IADL are potentially modifiable [26]. Our findings, therefore, have substantial implications for populations that are planning appropriate surveillance and intervention strategies for an ageing population.

Although its importance is widely recognized [2, 5, 19, 20, 21, 26], there is still no globally accepted gold standard for assessing PF [19, 20, 21]. Based on the number of studies, the GS appears to be the most frequently used test [1, 5, 9], widely applied as either a primary endpoint in randomized controlled trials or as the reference approach for comparing different physical performance indicators. Some international guidelines recommend GS and CST as interchangeable approaches for assessing PF [20, 21]. Other studies have tested both methods as complementary to each other [9]. To date, direct large‐scale comparative evidence on the superiority of GS versus CST remains scarce. A previous study reviewed the association between objectively measured physical activity and several clinical outcomes in community‐dwelling older adults, with the highest standardized effect size observed for the association between physical activity and CST‐indicated lower body muscle strength [2]. Although GS was not evaluated in this study, these results partially support our findings, suggesting that CST might outperform GS in predicting ADL/IADL by acting as a better indicator of physical activity (such as duration, intensity and frequency) [34]. A meta‐analysis also found that multicomponent training outperformed other training modalities in improving body composition (e.g., fat‐free mass) and GS, while resistance training was more beneficial for the 30‐s CST and handgrip strength [35]. These results suggest that GS is more strongly correlated with skeletal muscle volume and morphology pathways, as they change in similar patterns following intervention. In contrast, CST better represents muscle function, and more importantly, this representation extends beyond just the lower limbs. These results are in line with our findings, which showed that CST outperforms GS in FC items that typically do not involve lower limb muscles, such as eating and lifting arms. Contrary to our findings, one study conducted in older adults with cancer showed that the four‐meter GS exhibited stronger associations with dependence in one or more IADLs compared to CST [36]. However, the results of this study may be limited by its small sample size (*n* = 475) and the lack of statistical testing for differences in the AUC metrics. Nonetheless, our findings, combined with existing evidence [35], have the potential to inspire new individualized intervention strategies, such as using specific physical training modalities to intervene in a specific ADL/IADL item.

Another underlying explanation for CST being superior to GS may stem from the methodologies of the tests [20, 32]. First, during GS measurements, individuals are typically asked to walk at their usual pace [5, 9], which makes it difficult to capture their maximum muscle performance. In contrast, during CST measurements, participants are generally instructed to complete the test as quickly as possible [2, 32], which is more likely to reveal the upper limits of their muscle capacity. In support of this assumption, a previous study found that the relationship between chair stands and GS (quantified by standardized regression coefficients β) strengthens with the intensity of GS (from usual pace, to dual‐task GS, to maximum speed GS) [37]. It would be valuable for future studies to clarify whether GS measured at maximum speed outperforms usual‐speed GS in predicting ADL/IADL‐related outcomes. Second, a participant's height (or leg length) may significantly influence GS results. For example, consider two individuals of different heights who take the same number of steps in a given time frame—the taller person will likely cover a greater distance. In this case, the GS and the muscle function it represents may be biased. Indeed, many anthropometric and functional metrics, such as body mass index, appendicular skeletal muscle mass index and handgrip strength [38], are adjusted for body height. Future research could focus on standardizing GS, establishing cut‐off points for different ethnicities/populations, and evaluating the impact of such adjustments.

There are several potential limitations and generalizability issues in this study that must be noted. First, Asian populations may have anthropometric and physical differences compared to their Western counterparts [20, 21]. Therefore, these results should be re‐evaluated in non‐Asian populations. Second, this study used a 2.5‐m GS test; thus, the observed results, including the developed GS cutoffs, need to be replicated using other GS protocols (e.g., 6‐m GS). However, a previous study found that the correlation between the 2.4‐m GS and the 10‐m GS was extremely high (*r* = 0.989, *p* < 0.01) [39], suggesting that our results may be generalized to scenarios using different test distances. Third, the causal relationship between PF and the outcomes observed in our study is based solely on statistical methods. Mechanism studies or randomized controlled trials would help further validate the robustness of our results. Future studies are needed to address these issues.

In conclusion, the findings of this nationwide study suggest that CST is a better objective measure of PF than GS, exhibiting stronger non‐causal and causal relationships with multidimensional functional outcomes. As individuals age, the superiority of CST over GS remains consistent. Furthermore, CST's ability to represent FC extends beyond lower limb muscle‐related items, making it a more suitable indicator for comprehensive assessment purposes. Future studies are warranted to confirm our findings in other ethnic groups and across different disease and health scenarios.

## Funding

This work was supported by the National Natural Science Foundation of China (82304131), the Natural Science Foundation of Chongqing, China (CSTB2024NSCQ‐MSX1233), the Young Doctoral Talent Incubation Program of the Xinqiao Hospital, Army Medical University (2024YQB033), the Key Program of the Joint Funds of the National Natural Science Foundation of China (U22A20279) and the Key Project of Chongqing Technology Development and Application Program (CSTB2023TIAD‐KPX0060).

## Ethics Statement

The authors certify that the ethical guidelines for publishing of the *Journal of Cachexia, Sarcopenia and Muscle:* update 2019 have been followed [40]. National and international research ethics guidelines were followed, including the Deontological Code of Ethics and the 1964 Declaration of Helsinki and its later amendments. All patients provide written consent for the use of their data, and the study protocol of CHARLS was approved by the Ethical Review Committee of Peking University (approval number: IRB00001052‐11015).

## Conflicts of Interest

The authors declare no conflicts of interest.

## Supporting information


**Table S1:** Functional capacity items included for analysis in the present study.
**Table S2:** Supplemental methods on the double machine learning causal inference used in the present study.
**Table S3:** Association of objectively measured physical function with different factors in the 2013 wave.
**Table S4:** Association of objectively measured physical function with different factors in the 2015 wave.
**Table S5:** Diagnostic performance of objective physical function measures on multidimensional functional capacity in the 2013 wave.
**Table S6:** Diagnostic performance of objective physical function measures on multidimensional functional capacity in the 2015 wave.
**Table S7:** Association of objectively measured physical function stratified by optimized cutoffs with different factors in the 2011 wave.
**Table S8:** Association of objectively measured physical function stratified by optimized cutoffs with different factors in the 2013 wave.
**Table S9:** Association of objectively measured physical function stratified by optimized cutoffs with different factors in the 2015 wave.
**Figure S1:** Distribution of gait speed and chair stand test, stratified by functional capacity items.
**Figure S2:** Restricted cubic spline (RCS) analysis of the association between gait speed and the chair stand test with 20 functional capacity outcomes in the 2013 wave. (A) Gait speed and outcomes. (B) Chair stand test and outcomes.
**Figure S3:** Restricted cubic spline (RCS) analysis of the association between gait speed and the chair stand test with 20 functional capacity outcomes in the 2015 wave. (A) Gait speed and outcomes. (B) Chair stand test and outcomes.
**Figure S4:** Univariate and multivariate logistic regression analyses of the association between standardized gait speed and the chair stand test with 20 functional capacity outcomes in the 2013 wave. ADL, activities of daily living; IADL, instrumental activities of daily living; SD, standard deviation.
**Figure S5:** Univariate and multivariate logistic regression analyses of the association between standardized gait speed and the chair stand test with 20 functional capacity outcomes in the 2015 wave. ADL, activities of daily living; IADL, instrumental activities of daily living; SD, standard deviation.
**Figure S6:** Causal inference analysis comparing the causal effects of gait speed and the chair stand test on overall functional capacity at the global (population) and local (individual) levels. All models include age, sex, body mass index (BMI), gait speed (GS) and chair stand test (CST) as covariates. ATE, average treatment effect; TE, treatment effect. (A) Global causal effect in the baseline survey. (B) Local causal effect in the baseline survey. (C) Global causal effect in follow‐up wave one. (D) Local causal effect in follow‐up wave one. (E) Global causal effect in follow‐up wave two. (F) Local causal effect in follow‐up wave two.
