# Supplementary material for: Objective Measures of Physical Functioning, Disabilities in Daily Life and Trends During Ageing: A Repeated Cross‐Sectional Study
Source: J Cachexia Sarcopenia Muscle. 2025 Nov 23;16(6):e70133. doi: 10.1002/jcsm.70133 (PMC12641453; doi:10.1002/jcsm.70133)
Supplement: Supplementary file 1 — Table S1: Functional capacity items included for analysis in the present study. Table S2: Supplemental methods on the double machine learning causal inference used in the present study. Table S3: Association of objectively measured physical function with different factors in the 2013 wave. Table S4: Association of objectively measured physical function with different factors in the 2015 wave. Table S5: Diagnostic performance of objective physical function measures on multidimensional functional capacity in the 2013 wave. Table S6: Diagnostic performance of objective physical function measures on multidimensional functional capacity in the 2015 wave. Table S7: Association of objectively measured physical function stratified by optimized cutoffs with different factors in the 2011 wave. Table S8: Association of objectively measured physical function stratified by optimized cutoffs with different factors in the 2013 wave. Table S9: Association of objectively measured physical function stratified by optimized cutoffs with different factors in the 2015 wave. Figure S1: Distribution of gait speed and chair stand test, stratified by functional capacity items. Figure S2: Restricted cubic spline (RCS) analysis of the association between gait speed and the chair stand test with 20 functional capacity outcomes in the 2013 wave. (A) Gait speed and outcomes. (B) Chair stand test and outcomes. Figure S3: Restricted cubic spline (RCS) analysis of the association between gait speed and the chair stand test with 20 functional capacity outcomes in the 2015 wave. (A) Gait speed and outcomes. (B) Chair stand test and outcomes. Figure S4: Univariate and multivariate logistic regression analyses of the association between standardized gait speed and the chair stand test with 20 functional capacity outcomes in the 2013 wave. ADL, activities of daily living; IADL, instrumental activities of daily living; SD, standard deviation. Figure S5: Univariate and multivariate logistic regression anal [file JCSM-16-e70133-s001.pdf]

Supplementary files

Number	Description
1	**Table S1.** Functional capacity items included for analysis in the present study
2	**Table S2.** Supplemental methods on the double machine learning causal inference used in the present study
3	**Table S3.** Association of objectively measured physical function with different factors in the 2013 wave
4	**Table S4.** Association of objectively measured physical function with different factors in the 2015 wave
5	**Table S5.** Diagnostic performance of objective physical function measures on multidimensional functional capacity in the 2013 wave
6	**Table S6.** Diagnostic performance of objective physical function measures on multidimensional functional capacity in the 2015 wave
7	**Table S7.** Association of objectively measured physical function stratified by optimized cutoffs with different factors in the 2011 wave
8	**Table S8.** Association of objectively measured physical function stratified by optimized cutoffs with different factors in the 2013 wave
9	**Table S9.** Association of objectively measured physical function stratified by optimized cutoffs with different factors in the 2015 wave
10	**Figure S1.** Distribution of gait speed and chair stand test, stratified by functional capacity items.
11	**Figure S2.** Restricted cubic spline (RCS) analysis of the association between gait speed and the chair stand test with 20 functional capacity outcomes in the 2013 wave. (A) Gait speed and outcomes. (B) Chair stand test and outcomes.
12	**Figure S3.** Restricted cubic spline (RCS) analysis of the association between gait speed and the chair stand test with 20 functional capacity outcomes in the 2015 wave. (A) Gait speed and outcomes. (B) Chair stand test and outcomes.
13	**Figure S4.** Univariate and multivariate logistic regression analyses of the association between standardized gait speed and the chair stand test with 20 functional capacity outcomes in the 2013 wave. ADL, activities of daily living; IADL, instrumental activities of daily living; SD, standard deviation.
14	**Figure S5.** Univariate and multivariate logistic regression analyses of the association between standardized gait speed and the chair stand test with 20 functional capacity outcomes in the 2015 wave. ADL, activities of daily living; IADL, instrumental activities of daily living; SD, standard deviation.
15	**Figure S6.** Causal inference analysis comparing the causal effects of gait speed and the chair stand test on overall functional capacity at the global (population) and local (individual) levels. All models include age, sex, body mass index (BMI), gait speed (GS), and chair stand test (CST) as covariates. ATE, average treatment effect; TE, treatment effect. (A) Global causal effect in the baseline survey. (B) Local causal effect in the baseline survey. (C) Global causal effect in follow-up wave one. (D) Local causal effect in follow-up wave one. (E) Global causal effect in follow-up wave two. (F) Local causal effect in follow-up wave two.

Table S1. Functional capacity indices included for analysis in the present study

Category	Index abbreviation	Detailed description
ADL	Dressing	Do you have some difficulty with dressing?
	Bathing	Do you have some difficulty with bathing?
	Eating	Do you have some difficulty with eating?
	Bed	Do you have some difficulty with getting in and out of bed?
	Toilet	Do you have some difficulty with using the toilet?
	Urination	Do you have some difficulty with controlling urination and defecation?
IADL	Money	Do you have some difficulty with managing money?
	Medication	Do you have some difficulty with taking medications?
	Shopping	Do you have some difficulty with shopping for groceries?
	Meal	Do you have some difficulty with preparing meals?
	Housework	Do you have some difficulty with cleaning house?
Other	Jogging 1km	Do you have some difficulty with running or jogging 1km?
	Walking 1km	Do you have some difficulty with walking 1km?
	Walking 100m	Do you have some difficulty with walking 100m?
	Chair	Do you have some difficulty with getting up from a chair after sitting for long periods?
	Climbing	Do you have some difficulty with climbing several flights of stairs without resting?
	Stooping	Do you have some difficulty with stooping, kneeling, or crouching?
	Lifting 5kg	Do you have some difficulty with lifting or carrying weights over 5kg?
	Picking	Do you have some difficulty with picking up a coin from the table?
	Arm	Do you have some difficulty with reaching arms above shoulder level?

Abbreviations: ADL, activities of daily living; IADL, instrumental activities of daily living; Other, other functional capacity items.

Table S2. Supplemental methods on the double machine learning causal inference used in the present study

Item/definition	Description
Method summary	The impact of physical function on the physical capacity summary score was estimated using a double machine learning causal inference framework to obtain the importance of each feature. The method uses machine learning methods to identify the part of the observed outcome and treatment that is not predictable by the controls X, W (aka residual outcome and residual treatment). Then estimates a Conditional Average Treatment Effect (CATE) model by regressing the residual outcome on the residual treatment in a manner that accounts for heterogeneity in the regression coefficient, with respect to X.
Input/treatment variables	Objectively measured physical function (gait speed and 5-time chair stand test, standardized using a z-score approach to make the results comparable), age, sex, body mass index.
Outcome variable	The summary score of physical capacity in 20 dimensions.
Nuisance model	Separate nuisance models are trained to predict the outcome and also each individual input feature column from all of the other columns in the dataset as a prerequisite step before computing the actual causal effect for that feature column. Here, the light gradient boosting machine (LGBM) model is used with 5-fold cross-validation.
Heterogeneous model	A Light Gradient Boosting Machine (LGBM) model is trained to compute the estimated treatment effect for a input feature.

Table S3. Association of objectively measured physical function with different factors in the 2013 wave

		Gait speed (normal: ≥1m/s)			Five-time chair stand test (normal: <12s)		
n	Overall (n=4922)	Normal (n=349)	Impaired (n=4573)	*P*	Normal (n=3323)	Impaired (n=1599)	*P*
Age, years	65.0 [62.0, 71.0]	64.0 [61.0, 67.0]	66.0 [62.0, 71.0]	<0.001	65.0 [61.0, 69.0]	67.0 [63.0, 74.0]	<0.001
Sex, men	2525 (51.3)	242 (69.3)	2283 (49.9)	<0.001	1847 (55.6)	678 (42.4)	<0.001
Body mass index, kg/m^2	23.0 [20.6, 25.6]	23.6 [21.1, 25.8]	22.9 [20.6, 25.5]	0.017	23.1 [20.8, 25.6]	22.8 [20.2, 25.6]	0.071
Body mass index category				0.017			<0.001
I underweight	395 (8.0)	20 (5.7)	375 (8.2)		219 (6.6)	176 (11.0)	
II normal	2595 (52.7)	171 (49.0)	2424 (53.0)		1790 (53.9)	805 (50.3)	
III overweight	1418 (28.8)	125 (35.8)	1293 (28.3)		993 (29.9)	425 (26.6)	
IV obese	514 (10.4)	33 (9.5)	481 (10.5)		321 (9.7)	193 (12.1)	
Body height, m	1.6 [1.5, 1.6]	1.6 [1.6, 1.7]	1.6 [1.5, 1.6]	<0.001	1.6 [1.5, 1.6]	1.6 [1.5, 1.6]	<0.001
Body weight, kg	56.5 [49.8, 64.4]	60.8 [53.3, 67.7]	56.2 [49.6, 64.1]	<0.001	57.0 [50.5, 64.7]	55.5 [47.9, 63.8]	<0.001
Gait speed, m/s	0.7 [0.6, 0.8]	1.1 [1.0, 1.2]	0.7 [0.5, 0.8]	<0.001	0.7 [0.6, 0.9]	0.6 [0.5, 0.7]	<0.001
Five-time chair stand test, s	10.3 [8.4, 12.8]	8.3 [7.2, 9.9]	10.5 [8.6, 13.0]	<0.001	9.1 [7.7, 10.4]	14.2 [12.9, 16.7]	<0.001
ADL							
Dressing	228 (4.6)	10 (2.9)	218 (4.8)	0.134	113 (3.4)	115 (7.2)	<0.001
Bathing	269 (5.5)	10 (2.9)	259 (5.7)	0.036	115 (3.5)	154 (9.6)	<0.001
Eating	96 (2.0)	4 (1.1)	92 (2.0)	0.354	46 (1.4)	50 (3.1)	<0.001
Bed	238 (4.8)	10 (2.9)	228 (5.0)	0.099	123 (3.7)	115 (7.2)	<0.001
Toilet	628 (12.8)	23 (6.6)	605 (13.2)	<0.001	318 (9.6)	310 (19.4)	<0.001
Urination	215 (4.4)	5 (1.4)	210 (4.6)	0.008	112 (3.4)	103 (6.4)	<0.001
IADL							
Money	524 (10.6)	17 (4.9)	507 (11.1)	<0.001	279 (8.4)	245 (15.3)	<0.001
Medication	221 (4.5)	6 (1.7)	215 (4.7)	0.014	119 (3.6)	102 (6.4)	<0.001
Shopping	368 (7.5)	7 (2.0)	361 (7.9)	<0.001	175 (5.3)	193 (12.1)	<0.001
Meal	397 (8.1)	14 (4.0)	383 (8.4)	0.005	168 (5.1)	229 (14.3)	<0.001
Housework	501 (10.2)	12 (3.4)	489 (10.7)	<0.001	231 (7.0)	270 (16.9)	<0.001
Other function capacity items							
Jogging 1km	2968 (60.3)	152 (43.6)	2816 (61.6)	<0.001	1790 (53.9)	1178 (73.7)	<0.001

Walking 1km	865 (17.6)	24 (6.9)	841 (18.4)	<0.001	421 (12.7)	444 (27.8)	<0.001
Walking 100m	227 (4.6)	2 (0.6)	225 (4.9)	<0.001	100 (3.0)	127 (7.9)	<0.001
Chair	1481 (30.1)	78 (22.3)	1403 (30.7)	0.001	858 (25.8)	623 (39.0)	<0.001
Climbing	2216 (45.0)	102 (29.2)	2114 (46.2)	<0.001	1309 (39.4)	907 (56.7)	<0.001
Stooping	1753 (35.6)	74 (21.2)	1679 (36.7)	<0.001	996 (30.0)	757 (47.3)	<0.001
Lifting 5kg	703 (14.3)	22 (6.3)	681 (14.9)	<0.001	351 (10.6)	352 (22.0)	<0.001
Picking	202 (4.1)	8 (2.3)	194 (4.2)	0.103	122 (3.7)	80 (5.0)	0.033
Arm	519 (10.5)	20 (5.7)	499 (10.9)	0.003	286 (8.6)	233 (14.6)	<0.001

Abbreviations: ADL, activities of daily living; IADL, instrumental activities of daily living; Other, other functional capacity items.

Table S4. Association of objectively measured physical function with different factors in the 2015 wave

		Gait speed (normal: ≥1m/s)			Five-time chair stand test (normal: <12s)		
n	Overall (n=6165)	Normal (n=924)	Impaired (n=5241)	*P*	Normal (n=4637)	Impaired (n=1528)	*P*
Age, years	66.0 [62.0, 71.0]	64.0 [62.0, 68.0]	66.0 [62.0, 72.0]	<0.001	65.0 [62.0, 70.0]	69.0 [64.0, 75.0]	<0.001
Sex, men	3095 (50.2)	591 (64.0)	2504 (47.8)	<0.001	2485 (53.6)	610 (39.9)	<0.001
Body mass index, kg/m^2	23.1 [20.7, 25.6]	23.0 [20.9, 25.3]	23.1 [20.6, 25.7]	0.620	23.1 [20.7, 25.6]	23.1 [20.5, 25.7]	0.741
Body mass index category				0.106			<0.001
I underweight	500 (8.1)	57 (6.2)	443 (8.5)		338 (7.3)	162 (10.6)	
II normal	3194 (51.8)	491 (53.1)	2703 (51.6)		2435 (52.5)	759 (49.7)	
III overweight	1827 (29.6)	284 (30.7)	1543 (29.4)		1415 (30.5)	412 (27.0)	
IV obese	644 (10.4)	92 (10.0)	552 (10.5)		449 (9.7)	195 (12.8)	
Body height, m	1.6 [1.5, 1.6]	1.6 [1.5, 1.7]	1.6 [1.5, 1.6]	<0.001	1.6 [1.5, 1.6]	1.5 [1.5, 1.6]	<0.001
Body weight, kg	56.6 [49.7, 64.6]	58.6 [52.2, 66.9]	56.2 [49.3, 64.2]	<0.001	56.8 [50.2, 64.7]	55.7 [48.1, 64.3]	<0.001
Gait speed, m/s	0.8 [0.6, 0.9]	1.1 [1.0, 1.2]	0.7 [0.6, 0.8]	<0.001	1.2 [1.1, 1.5]	1.5 [1.3, 1.9]	<0.001
Five-time chair stand test, s	9.6 [7.8, 12.0]	8.1 [6.6, 9.8]	9.9 [8.0, 12.3]	<0.001	8.7 [7.3, 10.1]	14.3 [12.9, 16.7]	<0.001
ADL							
Dressing	384 (6.2)	36 (3.9)	348 (6.6)	0.002	208 (4.5)	176 (11.5)	<0.001
Bathing	471 (7.6)	32 (3.5)	439 (8.4)	<0.001	244 (5.3)	227 (14.9)	<0.001
Eating	147 (2.4)	9 (1.0)	138 (2.6)	0.003	74 (1.6)	73 (4.8)	<0.001
Bed	480 (7.8)	36 (3.9)	444 (8.5)	<0.001	272 (5.9)	208 (13.6)	<0.001
Toilet	959 (15.6)	94 (10.2)	865 (16.5)	<0.001	562 (12.1)	397 (26.0)	<0.001
Urination	321 (5.2)	32 (3.5)	289 (5.5)	0.012	196 (4.2)	125 (8.2)	<0.001
IADL							
Money	722 (11.7)	60 (6.5)	662 (12.6)	<0.001	430 (9.3)	292 (19.1)	<0.001
Medication	311 (5.0)	25 (2.7)	286 (5.5)	0.001	184 (4.0)	127 (8.3)	<0.001
Shopping	534 (8.7)	33 (3.6)	501 (9.6)	<0.001	264 (5.7)	270 (17.7)	<0.001
Meal	606 (9.8)	36 (3.9)	570 (10.9)	<0.001	311 (6.7)	295 (19.3)	<0.001
Housework	878 (14.2)	62 (6.7)	816 (15.6)	<0.001	497 (10.7)	381 (24.9)	<0.001
Other function capacity items							
Jogging 1km	3742 (60.7)	427 (46.2)	3315 (63.3)	<0.001	2553 (55.1)	1189 (77.8)	<0.001

Walking 1km	1286 (20.9)	77 (8.3)	1209 (23.1)	<0.001	714 (15.4)	572 (37.4)	<0.001
Walking 100m	484 (7.9)	20 (2.2)	464 (8.9)	<0.001	250 (5.4)	234 (15.3)	<0.001
Chair	1988 (32.2)	207 (22.4)	1781 (34.0)	<0.001	1274 (27.5)	714 (46.7)	<0.001
Climbing	2887 (46.8)	305 (33.0)	2582 (49.3)	<0.001	1921 (41.4)	966 (63.2)	<0.001
Stooping	2299 (37.3)	230 (24.9)	2069 (39.5)	<0.001	1463 (31.6)	836 (54.7)	<0.001
Lifting 5kg	1013 (16.4)	68 (7.4)	945 (18.0)	<0.001	563 (12.1)	450 (29.5)	<0.001
Picking	332 (5.4)	27 (2.9)	305 (5.8)	<0.001	207 (4.5)	125 (8.2)	<0.001
Arm	770 (12.5)	80 (8.7)	690 (13.2)	<0.001	465 (10.0)	305 (20.0)	<0.001

Abbreviations: ADL, activities of daily living; IADL, instrumental activities of daily living; Other, other functional capacity items.

Table S5. Diagnostic performance of objective physical function measures on multidimensional functional capacity in the 2013 wave

Index	Gait speed, m/s		Five-time chair stand test, s		Between-model comparison (reference = gait speed)				
	AUC (95%CI)	Cutoff	AUC (95%CI)	Cutoff	*P*-AUC	cNRI (95%CI)	*P*-cNRI	IDI (95%CI)	*P*-IDI
ADL									
Dressing	0.620 (0.585-0.656)	0.71	0.652 (0.616-0.688)	11.08	0.088	0.310 (0.177-0.443)	<0.001	0.012 (0.003-0.020)	0.007
Bathing	0.659 (0.625-0.692)	0.66	0.686 (0.653-0.718)	10.41	0.130	0.340 (0.218-0.462)	<0.001	0.014 (0.003-0.025)	0.013
Eating	0.634 (0.577-0.691)	0.67	0.635 (0.575-0.694)	13.85	0.979	0.162 (−0.040-0.363)	0.116	0.001 (−0.002-0.004)	0.383
Bed	0.621 (0.585-0.657)	0.68	0.631 (0.596-0.666)	10.64	0.615	0.181 (0.051-0.311)	0.006	0.010 (0.002-0.018)	0.019
Toilet	0.607 (0.584-0.631)	0.67	0.641 (0.618-0.664)	11.21	0.006	0.223 (0.140-0.307)	<0.001	0.011 (0.004-0.018)	0.002
Urination	0.597 (0.560-0.634)	0.74	0.608 (0.571-0.645)	11.60	0.600	0.108 (−0.029-0.244)	0.123	2×10^{-4} (−0.004-0.004)	0.943
IADL									
Money	0.601 (0.575-0.626)	0.66	0.604 (0.577-0.630)	11.04	0.832	0.149 (0.059-0.240)	0.001	0.004 (-7×10^{-4}-0.010)	0.088
Medication	0.604 (0.568-0.640)	0.71	0.606 (0.569-0.643)	10.24	0.925	0.150 (0.015-0.285)	0.029	0.001 (−0.006-0.008)	0.745
Shopping	0.659 (0.629-0.688)	0.66	0.650 (0.619-0.681)	11.06	0.583	0.052 (−0.054-0.159)	0.334	0.004 (−0.006-0.013)	0.437
Meal	0.656 (0.626-0.685)	0.65	0.678 (0.650-0.707)	11.28	0.126	0.240 (0.138-0.343)	<0.001	0.017 (0.007-0.027)	0.001
Housework	0.643 (0.618-0.669)	0.70	0.661 (0.636-0.687)	12.07	0.185	0.157 (0.065-0.249)	0.001	0.007 (−0.002-0.016)	0.142
Other									
Jogging 1km	0.627 (0.611-0.643)	0.67	0.629 (0.613-0.645)	10.28	0.840	0.080 (0.023-0.137)	0.006	0.009 (0.003-0.015)	0.002
Walking 1km	0.670 (0.651-0.690)	0.69	0.661 (0.641-0.681)	10.38	0.395	0.049 (−0.024-0.122)	0.191	0.003 (−0.006-0.011)	0.581
Walking 100m	0.703 (0.669-0.737)	0.64	0.677 (0.640-0.714)	13.52	0.192	0.153 (0.020-0.287)	0.024	0.006 (−0.003-0.015)	0.211
Chair	0.576 (0.559-0.594)	0.66	0.602 (0.585-0.619)	10.94	0.005	0.155 (0.094-0.215)	<0.001	0.014 (0.010-0.019)	<0.001
Climbing	0.614 (0.598-0.629)	0.63	0.617 (0.601-0.633)	10.33	0.703	0.095 (0.039-0.152)	<0.001	0.011 (0.006-0.017)	<0.001
Stooping	0.601 (0.584-0.617)	0.62	0.617 (0.601-0.634)	10.34	0.071	0.164 (0.106-0.222)	<0.001	0.016 (0.011-0.021)	<0.001
Lifting 5kg	0.667 (0.645-0.688)	0.64	0.649 (0.626-0.671)	11.07	0.136	0.040 (−0.040-0.119)	0.331	0.002 (−0.008-0.012)	0.653
Picking	0.610 (0.570-0.651)	0.60	0.605 (0.567-0.643)	9.74	0.795	−0.037 (−0.178-0.104)	0.607	−0.007 (−0.017-0.002)	0.128
Arm	0.600 (0.575-0.626)	0.68	0.608 (0.582-0.634)	11.31	0.562	0.183 (0.092-0.274)	<0.001	0.005 (3×10^{-4}-0.011)	0.040

Abbreviations: ADL, activities of daily living; IADL, instrumental activities of daily living; Other, other functional capacity items.

Table S6. Diagnostic performance of objective physical function measures on multidimensional functional capacity in the 2015 wave

	Gait speed, m/s		Five-time chair stand test, s		Between-model comparison (reference = walking speed)				
Index	AUC (95%CI)	Cutoff	AUC (95%CI)	Cutoff	*P*-AUC	cNRI (95%CI)	*P*-cNRI	IDI (95%CI)	*P*-IDI
ADL									
Dressing	0.630 (0.600-0.660)	0.72	0.657 (0.627-0.686)	10.84	0.127	0.178 (0.075-0.281)	0.001	0.006 (-6×10^{-4}-0.012)	0.077
Bathing	0.669 (0.643-0.695)	0.65	0.689 (0.664-0.715)	10.84	0.171	0.171 (0.078-0.265)	0.000	0.013 (0.003-0.023)	0.012
Eating	0.691 (0.646-0.736)	0.62	0.704 (0.661-0.747)	9.63	0.622	0.092 (−0.071-0.256)	0.267	−0.002 (−0.012-0.007)	0.657
Bed	0.654 (0.628-0.679)	0.75	0.658 (0.633-0.683)	10.10	0.770	0.109 (0.016-0.202)	0.022	0.003 (−0.003-0.009)	0.314
Toilet	0.612 (0.592-0.632)	0.63	0.644 (0.625-0.664)	10.84	0.003	0.251 (0.183-0.320)	<0.001	0.017 (0.011-0.023)	<0.001
Urination	0.622 (0.591-0.654)	0.73	0.610 (0.577-0.644)	9.81	0.532	0.112 (-6×10^{-4}-0.224)	0.051	0.003 (-2×10^{-4}-0.006)	0.065
IADL									
Money	0.627 (0.605-0.649)	0.66	0.636 (0.615-0.658)	10.71	0.464	0.075 (−0.002-0.153)	0.057	0.001 (−0.005-0.007)	0.686
Medication	0.616 (0.583-0.648)	0.62	0.639 (0.608-0.670)	9.60	0.203	0.157 (0.043-0.271)	0.007	0.004 (2×10^{-4}-0.007)	0.038
Shopping	0.691 (0.667-0.716)	0.62	0.698 (0.673-0.722)	10.97	0.648	0.137 (0.048-0.225)	0.002	0.003 (−0.008-0.015)	0.587
Meal	0.684 (0.661-0.707)	0.74	0.694 (0.671-0.717)	10.03	0.427	0.146 (0.063-0.230)	0.001	0.015 (0.006-0.025)	0.002
Housework	0.660 (0.641-0.680)	0.76	0.662 (0.642-0.682)	10.03	0.898	0.067 (−0.005-0.138)	0.066	0.004 (−0.003-0.012)	0.275
Other									
Jogging 1km	0.633 (0.619-0.647)	0.76	0.650 (0.636-0.663)	9.60	0.042	0.087 (0.036-0.138)	0.001	0.013 (0.007-0.019)	<0.001
Walking 1km	0.683 (0.666-0.699)	0.74	0.684 (0.668-0.701)	10.05	0.848	0.044 (−0.018-0.105)	0.165	0.005 (−0.005-0.014)	0.331
Walking 100m	0.707 (0.682-0.732)	0.75	0.693 (0.668-0.718)	10.08	0.320	−0.112 (−0.205-−0.019)	0.018	−0.015 (−0.027-−0.004)	0.007
Chair	0.603 (0.588-0.618)	0.76	0.632 (0.617-0.647)	10.01	<0.001	0.209 (0.156-0.262)	<0.001	0.023 (0.018-0.029)	<0.001
Climbing	0.622 (0.608-0.636)	0.77	0.638 (0.625-0.652)	9.99	0.042	0.162 (0.112-0.212)	<0.001	0.019 (0.013-0.024)	<0.001
Stooping	0.616 (0.602-0.631)	0.77	0.643 (0.629-0.657)	10.00	0.001	0.194 (0.142-0.245)	<0.001	0.025 (0.019-0.031)	<0.001
Lifting 5kg	0.663 (0.645-0.682)	0.73	0.672 (0.653-0.690)	10.80	0.439	0.065 (−0.003-0.132)	0.060	0.007 (-6×10^{-4}-0.015)	0.070
Picking	0.615 (0.583-0.646)	0.77	0.622 (0.591-0.653)	10.77	0.696	0.107 (−0.004-0.218)	0.058	0.002 (−0.002-0.006)	0.242
Arm	0.616 (0.594-0.637)	0.73	0.627 (0.605-0.648)	9.60	0.349	0.075 (0.000-0.151)	0.050	0.005 (-8×10^{-4}-0.010)	0.095

Abbreviations: ADL, activities of daily living; IADL, instrumental activities of daily living; Other, other functional capacity items.

Table S7. Association of objectively measured physical function stratified by optimized cutoffs with different factors in the 2011 wave

		Gait speed (normal: ≥0.66m/s)			Five-time chair stand test (normal: <10.85s)		
Characteristics	Overall (n=4753)	Normal (n=2147)	Impaired (n=2606)	*P*	Normal (n=2532)	Impaired (n=2221)	*P*
Age, years	65.0 [62.0, 71.0]	64.0 [61.0, 69.0]	67.0 [62.0, 72.0]	<0.001	64.0 [61.0, 69.0]	67.0 [62.0, 73.0]	<0.001
Sex, men	2517 (53.0)	1268 (59.1)	1249 (47.9)	<0.001	1518 (60.0)	999 (45.0)	<0.001
Body mass index, kg/m^2	22.4 [20.1, 25.0]	22.7 [20.4, 25.1]	22.2 [20.0, 24.8]	<0.001	22.5 [20.2, 25.1]	22.3 [20.0, 24.9]	0.205
Body mass index category				<0.001			0.024
I underweight	481 (10.1)	189 (8.8)	292 (11.2)		234 (9.2)	247 (11.1)	
II normal	2660 (56.0)	1173 (54.6)	1487 (57.1)		1428 (56.4)	1232 (55.5)	
III overweight	1208 (25.4)	618 (28.8)	590 (22.6)		671 (26.5)	537 (24.2)	
IV obese	404 (8.5)	167 (7.8)	237 (9.1)		199 (7.9)	205 (9.2)	
Body height, m	1.6 [1.5, 1.6]	1.6 [1.5, 1.6]	1.6 [1.5, 1.6]	<0.001	1.6 [1.5, 1.6]	1.6 [1.5, 1.6]	<0.001
Body weight, kg	55.1 [48.4, 63.3]	56.6 [50.2, 64.5]	53.8 [47.1, 62.0]	<0.001	55.8 [49.4, 63.7]	54.1 [47.1, 62.6]	<0.001
Five-time chair stand test, s	10.6 [8.8, 13.3]	9.8 [8.0, 11.7]	11.6 [9.5, 14.4]	<0.001	8.9 [7.6, 9.9]	13.5 [12.1, 15.7]	<0.001
Walking time, 2.5m	3.9 [3.2, 5.0]	3.2 [2.8, 3.5]	4.8 [4.3, 6.0]	<0.001	3.6 [3.0, 4.5]	4.4 [3.6, 5.6]	<0.001
Walking speed, m/s	0.6 [0.5, 0.8]	0.8 [0.7, 0.9]	0.5 [0.4, 0.6]	<0.001	0.7 [0.6, 0.8]	0.6 [0.4, 0.7]	<0.001
ADL							
Dressing	188 (4.0)	46 (2.1)	142 (5.4)	<0.001	60 (2.4)	128 (5.8)	<0.001
Bathing	227 (4.8)	63 (2.9)	164 (6.3)	<0.001	64 (2.5)	163 (7.3)	<0.001
Eating	92 (1.9)	24 (1.1)	68 (2.6)	<0.001	18 (0.7)	74 (3.3)	<0.001
Bed	196 (4.1)	55 (2.6)	141 (5.4)	<0.001	66 (2.6)	130 (5.9)	<0.001
Toilet	589 (12.4)	181 (8.4)	408 (15.7)	<0.001	211 (8.3)	378 (17.0)	<0.001
Urination	216 (4.5)	61 (2.8)	155 (5.9)	<0.001	94 (3.7)	122 (5.5)	0.004
IADL							
Money	619 (13.0)	203 (9.5)	416 (16.0)	<0.001	224 (8.8)	395 (17.8)	<0.001
Medication	307 (6.5)	101 (4.7)	206 (7.9)	<0.001	106 (4.2)	201 (9.0)	<0.001
Shopping	368 (7.7)	97 (4.5)	271 (10.4)	<0.001	102 (4.0)	266 (12.0)	<0.001
Meal	339 (7.1)	104 (4.8)	235 (9.0)	<0.001	112 (4.4)	227 (10.2)	<0.001
Housework	371 (7.8)	108 (5.0)	263 (10.1)	<0.001	114 (4.5)	257 (11.6)	<0.001
Other							

Jogging 1km	2816 (59.2)	1086 (50.6)	1730 (66.4)	<0.001	1304 (51.5)	1512 (68.1)	<0.001
Walking 1km	536 (11.3)	136 (6.3)	400 (15.3)	<0.001	172 (6.8)	364 (16.4)	<0.001
Walking 100m	99 (2.1)	20 (0.9)	79 (3.0)	<0.001	25 (1.0)	74 (3.3)	<0.001
Chair	1303 (27.4)	463 (21.6)	840 (32.2)	<0.001	500 (19.7)	803 (36.2)	<0.001
Climbing	2092 (44.0)	782 (36.4)	1310 (50.3)	<0.001	893 (35.3)	1199 (54.0)	<0.001
Stooping	1482 (31.2)	548 (25.5)	934 (35.8)	<0.001	603 (23.8)	879 (39.6)	<0.001
Lifting 5kg	536 (11.3)	146 (6.8)	390 (15.0)	<0.001	177 (7.0)	359 (16.2)	<0.001
Picking	163 (3.4)	49 (2.3)	114 (4.4)	<0.001	56 (2.2)	107 (4.8)	<0.001
Arm	461 (9.7)	160 (7.5)	301 (11.6)	<0.001	164 (6.5)	297 (13.4)	<0.001

Abbreviations: ADL, activities of daily living; IADL, instrumental activities of daily living; Other, other functional capacity items.

Table S8. Association of objectively measured physical function stratified by optimized cutoffs with different factors in the 2013 wave

		Gait speed (normal: ≥0.66m/s)			Five-time chair stand test (normal: <10.85s)		
Characteristics	Overall (n=4922)	Normal (n=2729)	Impaired (n=2193)	*P*	Normal (n=2767)	Impaired (n=2155)	*P*
Age, years	65.0 [62.0, 71.0]	64.0 [61.0, 69.0]	67.0 [62.0, 73.0]	<0.001	64.0 [61.0, 69.0]	67.0 [63.0, 73.0]	<0.001
Sex, men	2525 (51.3)	1568 (57.5)	957 (43.6)	<0.001	1562 (56.5)	963 (44.7)	<0.001
Body mass index, kg/m^2	23.0 [20.6, 25.6]	23.2 [20.9, 25.6]	22.7 [20.3, 25.5]	<0.001	23.1 [20.8, 25.5]	22.8 [20.3, 25.6]	0.091
Body mass index category				<0.001			<0.001
I underweight	395 (8.0)	168 (6.2)	227 (10.4)		176 (6.4)	219 (10.2)	
II normal	2595 (52.7)	1427 (52.3)	1168 (53.3)		1495 (54.0)	1100 (51.0)	
III overweight	1418 (28.8)	867 (31.8)	551 (25.1)		835 (30.2)	583 (27.1)	
IV obese	514 (10.4)	267 (9.8)	247 (11.3)		261 (9.4)	253 (11.7)	
Body height, m	1.6 [1.5, 1.6]	1.6 [1.5, 1.6]	1.5 [1.5, 1.6]	<0.001	1.6 [1.5, 1.6]	1.6 [1.5, 1.6]	<0.001
Body weight, kg	56.5 [49.8, 64.4]	57.7 [51.4, 65.5]	55.0 [47.9, 63.1]	<0.001	57.0 [50.7, 64.8]	55.9 [48.1, 64.0]	<0.001
Gait speed, m/s	0.7 [0.6, 0.8]	0.8 [0.7, 0.9]	0.5 [0.5, 0.6]	<0.001	0.7 [0.6, 0.9]	0.6 [0.5, 0.7]	<0.001
Five-time chair stand test, s	10.3 [8.4, 12.8]	9.4 [7.9, 11.4]	11.8 [9.6, 14.6]	<0.001	8.7 [7.4, 9.7]	13.3 [11.9, 15.6]	<0.001
ADL							
Dressing	228 (4.6)	85 (3.1)	143 (6.5)	<0.001	77 (2.8)	151 (7.0)	<0.001
Bathing	269 (5.5)	83 (3.0)	186 (8.5)	<0.001	84 (3.0)	185 (8.6)	<0.001
Eating	96 (2.0)	33 (1.2)	63 (2.9)	<0.001	36 (1.3)	60 (2.8)	<0.001
Bed	238 (4.8)	90 (3.3)	148 (6.7)	<0.001	96 (3.5)	142 (6.6)	<0.001
Toilet	628 (12.8)	258 (9.5)	370 (16.9)	<0.001	236 (8.5)	392 (18.2)	<0.001
Urination	215 (4.4)	87 (3.2)	128 (5.8)	<0.001	85 (3.1)	130 (6.0)	<0.001
IADL							
Money	524 (10.6)	218 (8.0)	306 (14.0)	<0.001	221 (8.0)	303 (14.1)	<0.001
Medication	221 (4.5)	94 (3.4)	127 (5.8)	<0.001	93 (3.4)	128 (5.9)	<0.001
Shopping	368 (7.5)	124 (4.5)	244 (11.1)	<0.001	131 (4.7)	237 (11.0)	<0.001
Meal	397 (8.1)	134 (4.9)	263 (12.0)	<0.001	126 (4.6)	271 (12.6)	<0.001
Housework	501 (10.2)	187 (6.9)	314 (14.3)	<0.001	183 (6.6)	318 (14.8)	<0.001
Other							
Jogging 1km	2968 (60.3)	1417 (51.9)	1551 (70.7)	<0.001	1453 (52.5)	1515 (70.3)	<0.001

Walking 1km	865 (17.6)	302 (11.1)	563 (25.7)	<0.001	327 (11.8)	538 (25.0)	<0.001
Walking 100m	227 (4.6)	60 (2.2)	167 (7.6)	<0.001	86 (3.1)	141 (6.5)	<0.001
Chair	1481 (30.1)	700 (25.7)	781 (35.6)	<0.001	676 (24.4)	805 (37.4)	<0.001
Climbing	2216 (45.0)	1031 (37.8)	1185 (54.0)	<0.001	1042 (37.7)	1174 (54.5)	<0.001
Stooping	1753 (35.6)	800 (29.3)	953 (43.5)	<0.001	799 (28.9)	954 (44.3)	<0.001
Lifting 5kg	703 (14.3)	241 (8.8)	462 (21.1)	<0.001	265 (9.6)	438 (20.3)	<0.001
Picking	202 (4.1)	80 (2.9)	122 (5.6)	<0.001	87 (3.1)	115 (5.3)	<0.001
Arm	519 (10.5)	216 (7.9)	303 (13.8)	<0.001	214 (7.7)	305 (14.2)	<0.001

Abbreviations: ADL, activities of daily living; IADL, instrumental activities of daily living; Other, other functional capacity items.

Table S9. Association of objectively measured physical function stratified by optimized cutoffs with different factors in the 2015 wave

		Gait speed (normal: ≥0.66m/s)			Five-time chair stand test (normal: <10.85s)		
Characteristics	Overall (n=6165)	Normal (n=4356)	Impaired (n=1809)	*P*	Normal (n=4007)	Impaired (n=2158)	*P*
Age, years	66.0 [62.0, 71.0]	65.0 [62.0, 70.0]	69.0 [64.0, 76.0]	<0.001	65.0 [62.0, 70.0]	68.0 [64.0, 74.0]	<0.001
Sex, men	3095 (50.2)	2381 (54.7)	714 (39.5)	<0.001	2182 (54.5)	913 (42.3)	<0.001
Body mass index, kg/m^2	23.1 [20.7, 25.6]	23.2 [20.8, 25.6]	22.8 [20.2, 25.6]	<0.001	23.1 [20.7, 25.5]	23.1 [20.6, 25.7]	0.912
Body mass index category				<0.001			<0.001
I underweight	500 (8.1)	295 (6.8)	205 (11.3)		286 (7.1)	214 (9.9)	
II normal	3194 (51.8)	2264 (52.0)	930 (51.4)		2123 (53.0)	1071 (49.6)	
III overweight	1827 (29.6)	1364 (31.3)	463 (25.6)		1219 (30.4)	608 (28.2)	
IV obese	644 (10.4)	433 (9.9)	211 (11.7)		379 (9.5)	265 (12.3)	
Body height, m	1.6 [1.5, 1.6]	1.6 [1.5, 1.6]	1.5 [1.5, 1.6]	<0.001	1.6 [1.5, 1.6]	1.6 [1.5, 1.6]	<0.001
Body weight, kg	56.6 [49.7, 64.6]	57.7 [51.0, 65.5]	54.0 [47.1, 61.7]	<0.001	56.8 [50.3, 64.8]	56.0 [48.5, 64.2]	0.001
Gait speed, m/s	0.8 [0.6, 0.9]	0.9 [0.8, 1.0]	0.6 [0.5, 0.6]	<0.001	0.8 [0.7, 1.0]	0.7 [0.5, 0.8]	<0.001
Five-time chair stand test, s	9.6 [7.8, 12.0]	9.1 [7.4, 11.0]	11.3 [9.1, 14.6]	<0.001	8.3 [7.1, 9.5]	13.1 [11.8, 15.4]	<0.001
ADL							
Dressing	384 (6.2)	205 (4.7)	179 (9.9)	<0.001	158 (3.9)	226 (10.5)	<0.001
Bathing	471 (7.6)	218 (5.0)	253 (14.0)	<0.001	183 (4.6)	288 (13.3)	<0.001
Eating	147 (2.4)	63 (1.4)	84 (4.6)	<0.001	53 (1.3)	94 (4.4)	<0.001
Bed	480 (7.8)	240 (5.5)	240 (13.3)	<0.001	211 (5.3)	269 (12.5)	<0.001
Toilet	959 (15.6)	555 (12.7)	404 (22.3)	<0.001	450 (11.2)	509 (23.6)	<0.001
Urination	321 (5.2)	175 (4.0)	146 (8.1)	<0.001	161 (4.0)	160 (7.4)	<0.001
IADL							
Money	722 (11.7)	393 (9.0)	329 (18.2)	<0.001	334 (8.3)	388 (18.0)	<0.001
Medication	311 (5.0)	171 (3.9)	140 (7.7)	<0.001	150 (3.7)	161 (7.5)	<0.001
Shopping	534 (8.7)	239 (5.5)	295 (16.3)	<0.001	202 (5.0)	332 (15.4)	<0.001
Meal	606 (9.8)	286 (6.6)	320 (17.7)	<0.001	235 (5.9)	371 (17.2)	<0.001
Housework	878 (14.2)	460 (10.6)	418 (23.1)	<0.001	388 (9.7)	490 (22.7)	<0.001
Other							
Jogging 1km	3742 (60.7)	2382 (54.7)	1360 (75.2)	<0.001	2144 (53.5)	1598 (74.1)	<0.001

Walking 1km	1286 (20.9)	646 (14.8)	640 (35.4)	<0.001	559 (14.0)	727 (33.7)	<0.001
Walking 100m	484 (7.9)	203 (4.7)	281 (15.5)	<0.001	192 (4.8)	292 (13.5)	<0.001
Chair	1988 (32.2)	1222 (28.1)	766 (42.3)	<0.001	1044 (26.1)	944 (43.7)	<0.001
Climbing	2887 (46.8)	1796 (41.2)	1091 (60.3)	<0.001	1583 (39.5)	1304 (60.4)	<0.001
Stooping	2299 (37.3)	1415 (32.5)	884 (48.9)	<0.001	1211 (30.2)	1088 (50.4)	<0.001
Lifting 5kg	1013 (16.4)	534 (12.3)	479 (26.5)	<0.001	447 (11.2)	566 (26.2)	<0.001
Picking	332 (5.4)	188 (4.3)	144 (8.0)	<0.001	157 (3.9)	175 (8.1)	<0.001
Arm	770 (12.5)	435 (10.0)	335 (18.5)	<0.001	387 (9.7)	383 (17.7)	<0.001

Abbreviations: ADL, activities of daily living; IADL, instrumental activities of daily living; Other, other functional capacity items.

Figure S1

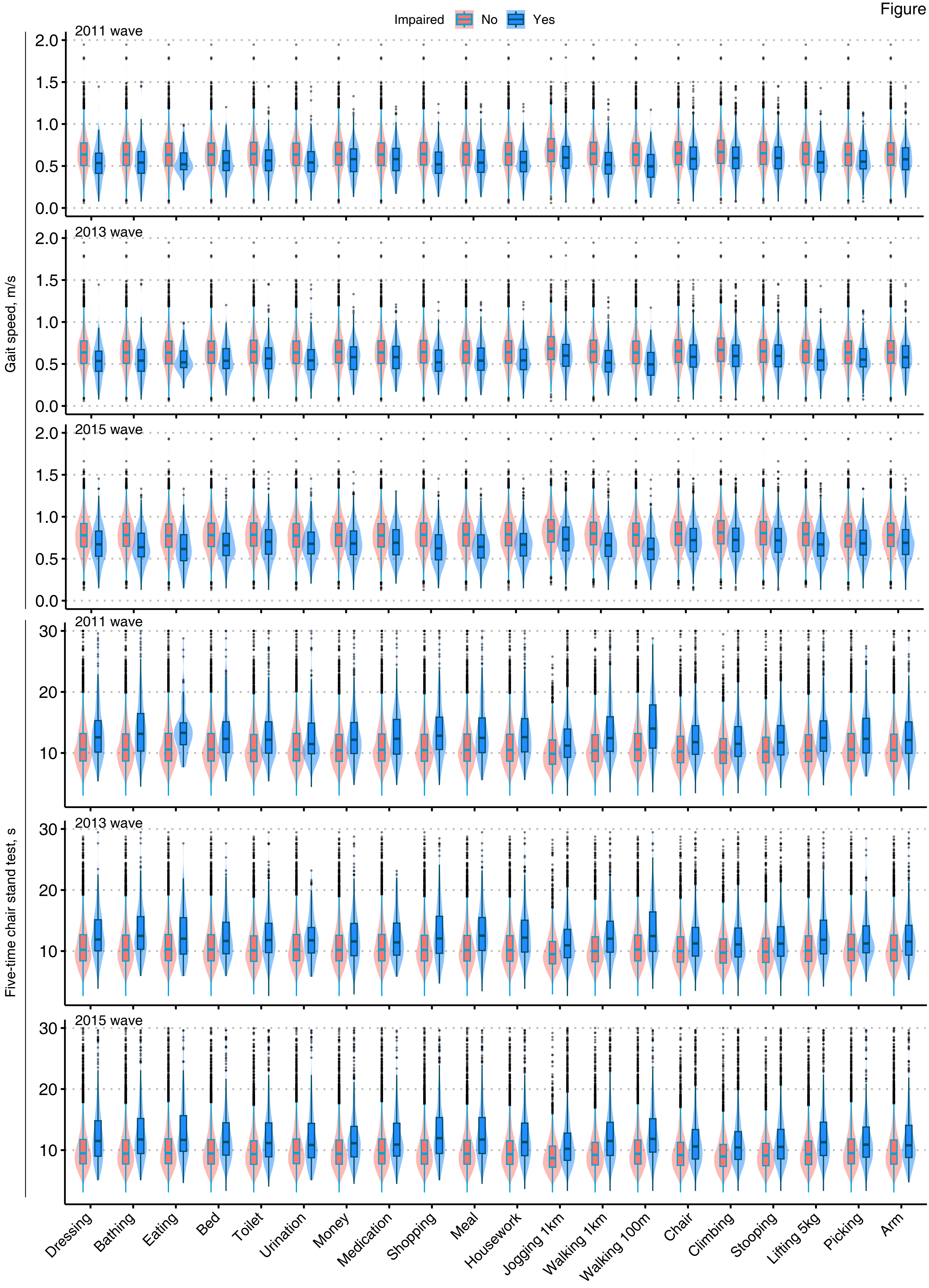

Impaired No Yes
2011 wave
2013 wave
2015 wave
Gait speed, m/s
Five-time chair stand test, s
0.0
0.5
1.0
1.5
2.0
10
20
30
Dressing
Bathing
Eating
Bed
Toilet
Urination
Money
Medication
Shopping
Meal
Housework
Jogging 1km
Walking 1km
Walking 100m
Chair
Climbing
Stooping
Lifting 5kg
Picking
Arm

Figure S2

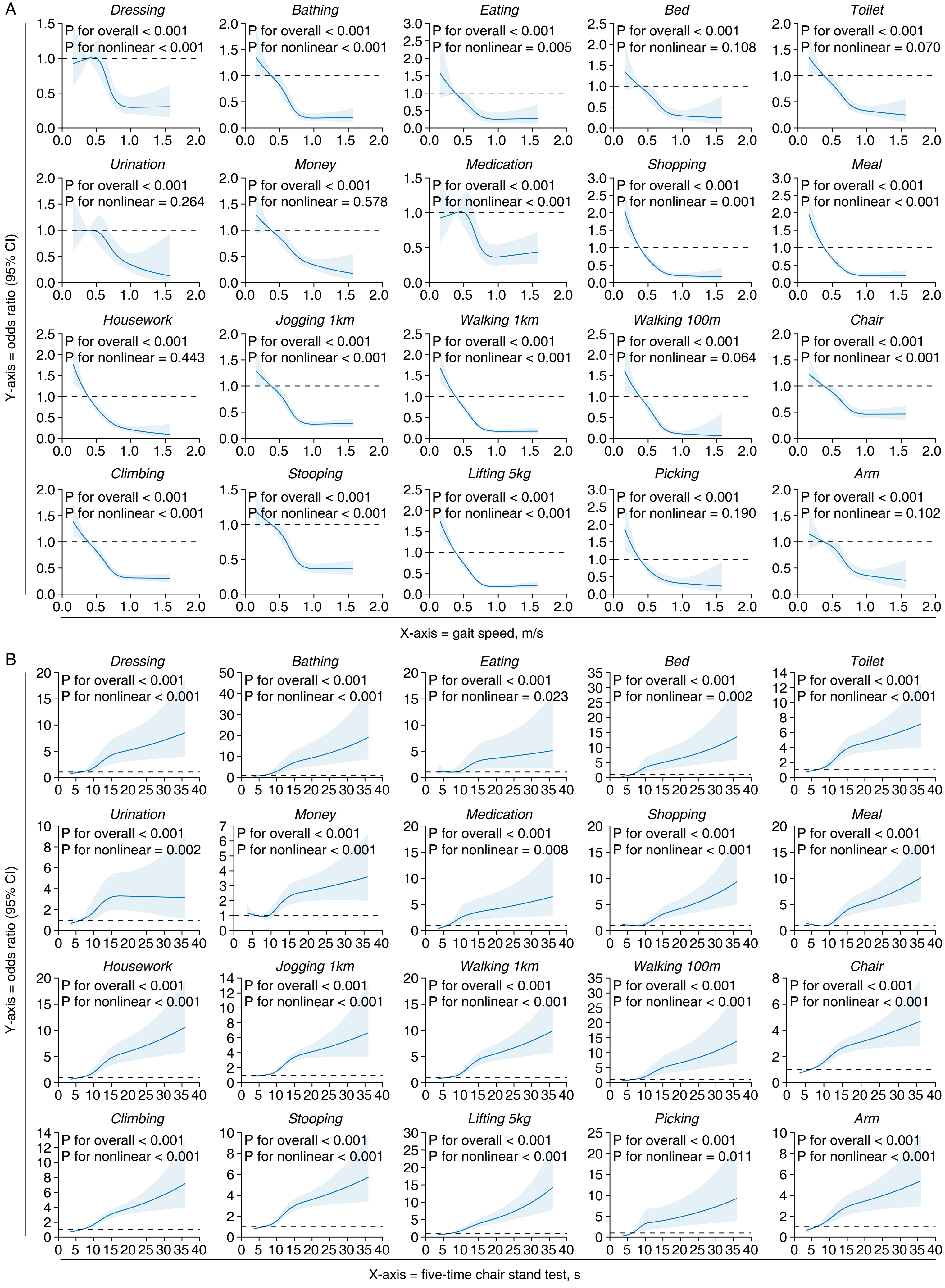
A
Dressing
P for overall < 0.001
P for nonlinear < 0.001
Bathing
P for overall < 0.001
P for nonlinear < 0.001
Eating
P for overall < 0.001
P for nonlinear = 0.005
Bed
P for overall < 0.001
P for nonlinear = 0.108
Toilet
P for overall < 0.001
P for nonlinear = 0.070
Urination
P for overall < 0.001
P for nonlinear = 0.264
Money
P for overall < 0.001
P for nonlinear = 0.578
Medication
P for overall < 0.001
P for nonlinear < 0.001
Shopping
P for overall < 0.001
P for nonlinear = 0.001
Meal
P for overall < 0.001
P for nonlinear < 0.001
Housework
P for overall < 0.001
P for nonlinear = 0.443
Jogging 1km
P for overall < 0.001
P for nonlinear < 0.001
Walking 1km
P for overall < 0.001
P for nonlinear < 0.001
Walking 100m
P for overall < 0.001
P for nonlinear = 0.064
Chair
P for overall < 0.001
P for nonlinear < 0.001
Climbing
P for overall < 0.001
P for nonlinear < 0.001
Stooping
P for overall < 0.001
P for nonlinear < 0.001
Lifting 5kg
P for overall < 0.001
P for nonlinear < 0.001
Picking
P for overall < 0.001
P for nonlinear = 0.190
Arm
P for overall < 0.001
P for nonlinear = 0.102
Y-axis = odds ratio (95% CI)
X-axis = gait speed, m/s
B
Dressing
P for overall < 0.001
P for nonlinear < 0.001
Bathing
P for overall < 0.001
P for nonlinear < 0.001
Eating
P for overall < 0.001
P for nonlinear = 0.023
Bed
P for overall < 0.001
P for nonlinear = 0.002
Toilet
P for overall < 0.001
P for nonlinear < 0.001
Urination
P for overall < 0.001
P for nonlinear = 0.002
Money
P for overall < 0.001
P for nonlinear < 0.001
Medication
P for overall < 0.001
P for nonlinear = 0.008
Shopping
P for overall < 0.001
P for nonlinear < 0.001
Meal
P for overall < 0.001
P for nonlinear < 0.001
Housework
P for overall < 0.001
P for nonlinear < 0.001
Jogging 1km
P for overall < 0.001
P for nonlinear < 0.001
Walking 1km
P for overall < 0.001
P for nonlinear < 0.001
Walking 100m
P for overall < 0.001
P for nonlinear < 0.001
Chair
P for overall < 0.001
P for nonlinear < 0.001
Climbing
P for overall < 0.001
P for nonlinear < 0.001
Stooping
P for overall < 0.001
P for nonlinear < 0.001
Lifting 5kg
P for overall < 0.001
P for nonlinear < 0.001
Picking
P for overall < 0.001
P for nonlinear = 0.011
Arm
P for overall < 0.001
P for nonlinear < 0.001
Y-axis = odds ratio (95% CI)
X-axis = five-time chair stand test, s

Figure S3

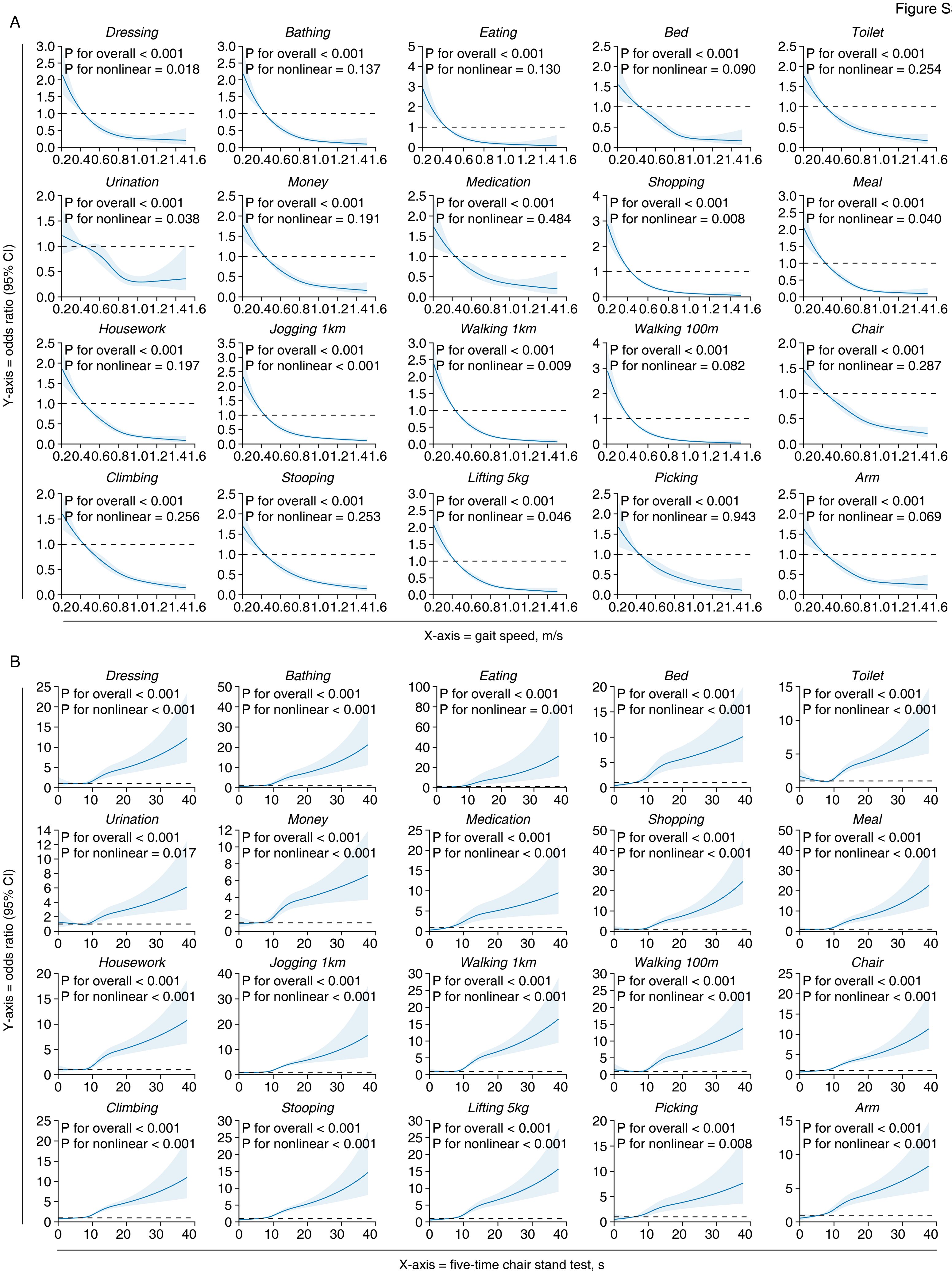
A
Dressing
P for overall < 0.001
P for nonlinear = 0.018
Bathing
P for overall < 0.001
P for nonlinear = 0.137
Eating
P for overall < 0.001
P for nonlinear = 0.130
Bed
P for overall < 0.001
P for nonlinear = 0.090
Toilet
P for overall < 0.001
P for nonlinear = 0.254
Urination
P for overall < 0.001
P for nonlinear = 0.038
Money
P for overall < 0.001
P for nonlinear = 0.191
Medication
P for overall < 0.001
P for nonlinear = 0.484
Shopping
P for overall < 0.001
P for nonlinear = 0.008
Meal
P for overall < 0.001
P for nonlinear = 0.040
Housework
P for overall < 0.001
P for nonlinear = 0.197
Jogging 1km
P for overall < 0.001
P for nonlinear < 0.001
Walking 1km
P for overall < 0.001
P for nonlinear = 0.009
Walking 100m
P for overall < 0.001
P for nonlinear = 0.082
Chair
P for overall < 0.001
P for nonlinear = 0.287
Climbing
P for overall < 0.001
P for nonlinear = 0.256
Stooping
P for overall < 0.001
P for nonlinear = 0.253
Lifting 5kg
P for overall < 0.001
P for nonlinear = 0.046
Picking
P for overall < 0.001
P for nonlinear = 0.943
Arm
P for overall < 0.001
P for nonlinear = 0.069
Y-axis = odds ratio (95% CI)
X-axis = gait speed, m/s
B
Dressing
P for overall < 0.001
P for nonlinear < 0.001
Bathing
P for overall < 0.001
P for nonlinear < 0.001
Eating
P for overall < 0.001
P for nonlinear = 0.001
Bed
P for overall < 0.001
P for nonlinear < 0.001
Toilet
P for overall < 0.001
P for nonlinear < 0.001
Urination
P for overall < 0.001
P for nonlinear = 0.017
Money
P for overall < 0.001
P for nonlinear < 0.001
Medication
P for overall < 0.001
P for nonlinear < 0.001
Shopping
P for overall < 0.001
P for nonlinear < 0.001
Meal
P for overall < 0.001
P for nonlinear < 0.001
Housework
P for overall < 0.001
P for nonlinear < 0.001
Jogging 1km
P for overall < 0.001
P for nonlinear < 0.001
Walking 1km
P for overall < 0.001
P for nonlinear < 0.001
Walking 100m
P for overall < 0.001
P for nonlinear < 0.001
Chair
P for overall < 0.001
P for nonlinear < 0.001
Climbing
P for overall < 0.001
P for nonlinear < 0.001
Stooping
P for overall < 0.001
P for nonlinear < 0.001
Lifting 5kg
P for overall < 0.001
P for nonlinear < 0.001
Picking
P for overall < 0.001
P for nonlinear = 0.008
Arm
P for overall < 0.001
P for nonlinear < 0.001
Y-axis = odds ratio (95% CI)
X-axis = five-time chair stand test, s

Figure S4

Model ■ Gait speed, m/s, per SD decreasing ◆ Five-time chair stand test, s, per SD increasing

Index	n/events	Crude OR (95%CI) Gait speed	Crude OR (95%CI) Chair stand test	Fully-adjusted OR (95%CI) Gait speed	Fully-adjusted OR (95%CI) Chair stand test
ADL					
Dressing	4922/228	1.188 (1.080-1.297)	1.402 (1.276-1.542)	1.137 (1.025-1.249)	1.356 (1.230-1.498)
Bathing	4922/269	1.324 (1.217-1.442)	1.549 (1.412-1.702)	1.272 (1.166-1.389)	1.491 (1.355-1.645)
Eating	4922/96	1.238 (1.097-1.374)	1.298 (1.145-1.457)	1.205 (1.057-1.348)	1.273 (1.113-1.437)
Bed	4922/238	1.225 (1.120-1.334)	1.380 (1.257-1.516)	1.170 (1.062-1.281)	1.320 (1.199-1.453)
Toilet	4922/628	1.304 (1.215-1.402)	1.466 (1.360-1.582)	1.236 (1.150-1.331)	1.386 (1.283-1.500)
Urination	4922/215	1.192 (1.082-1.302)	1.238 (1.120-1.363)	1.162 (1.049-1.276)	1.206 (1.085-1.334)
IADL					
Money	4922/524	1.239 (1.152-1.333)	1.317 (1.220-1.422)	1.205 (1.117-1.299)	1.282 (1.185-1.387)
Medication	4922/221	1.214 (1.106-1.324)	1.272 (1.155-1.398)	1.149 (1.034-1.262)	1.200 (1.079-1.325)
Shopping	4922/368	1.438 (1.325-1.562)	1.515 (1.391-1.652)	1.357 (1.249-1.478)	1.416 (1.297-1.548)
Meal	4922/397	1.400 (1.294-1.518)	1.606 (1.475-1.752)	1.325 (1.221-1.441)	1.527 (1.396-1.674)
Housework	4922/501	1.424 (1.319-1.539)	1.537 (1.419-1.667)	1.351 (1.249-1.463)	1.453 (1.338-1.581)
Other					
Jogging 1km	4922/2968	1.632 (1.504-1.776)	1.687 (1.564-1.822)	1.421 (1.310-1.546)	1.514 (1.401-1.638)
Walking 1km	4922/865	1.607 (1.493-1.732)	1.628 (1.513-1.753)	1.464 (1.358-1.581)	1.470 (1.363-1.587)
Walking 100m	4922/227	1.387 (1.271-1.518)	1.499 (1.362-1.652)	1.297 (1.185-1.422)	1.380 (1.252-1.524)
Chair	4922/1481	1.229 (1.156-1.309)	1.394 (1.308-1.488)	1.186 (1.114-1.265)	1.353 (1.266-1.447)
Climbing	4922/2216	1.446 (1.350-1.553)	1.550 (1.449-1.660)	1.328 (1.238-1.427)	1.438 (1.342-1.543)
Stooping	4922/1753	1.345 (1.260-1.437)	1.502 (1.407-1.606)	1.278 (1.197-1.368)	1.436 (1.343-1.539)
Lifting 5kg	4922/703	1.562 (1.449-1.685)	1.604 (1.487-1.732)	1.387 (1.284-1.500)	1.398 (1.294-1.513)
Picking	4922/202	1.321 (1.209-1.445)	1.288 (1.167-1.419)	1.271 (1.158-1.394)	1.223 (1.101-1.353)
Arm	4922/519	1.232 (1.146-1.326)	1.334 (1.236-1.441)	1.207 (1.120-1.301)	1.309 (1.210-1.419)

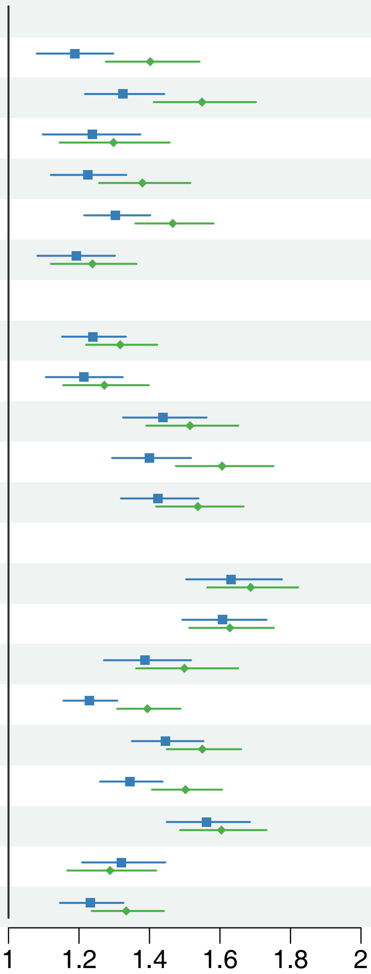

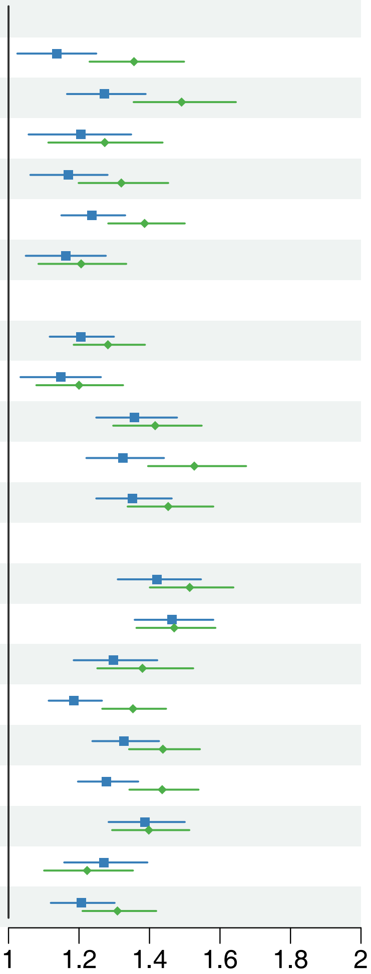

Figure S5

Model — Gait speed, m/s, per SD decreasing — Five-time chair stand test, s, per SD increasing

Index	n/events	Crude OR (95%CI) Gait speed	Crude OR (95%CI) Chair stand test	Fully-adjusted OR (95%CI) Gait speed	Fully-adjusted OR (95%CI) Chair stand test
ADL					
Dressing	6165/384	1.420 (1.316-1.532)	1.506 (1.395-1.625)	1.387 (1.280-1.502)	1.480 (1.366-1.604)
Bathing	6165/471	1.528 (1.422-1.643)	1.648 (1.531-1.775)	1.472 (1.364-1.590)	1.599 (1.480-1.729)
Eating	6165/147	1.542 (1.399-1.697)	1.558 (1.414-1.716)	1.504 (1.357-1.663)	1.524 (1.376-1.686)
Bed	6165/480	1.427 (1.329-1.533)	1.465 (1.363-1.575)	1.352 (1.253-1.459)	1.395 (1.294-1.505)
Toilet	6165/959	1.367 (1.286-1.453)	1.533 (1.440-1.634)	1.281 (1.201-1.367)	1.457 (1.365-1.557)
Urination	6165/321	1.294 (1.189-1.403)	1.347 (1.240-1.459)	1.211 (1.101-1.325)	1.273 (1.165-1.387)
IADL					
Money	6165/722	1.411 (1.322-1.506)	1.435 (1.344-1.532)	1.369 (1.277-1.468)	1.396 (1.303-1.496)
Medication	6165/311	1.310 (1.204-1.421)	1.387 (1.278-1.503)	1.243 (1.133-1.357)	1.335 (1.223-1.454)
Shopping	6165/534	1.701 (1.583-1.829)	1.738 (1.616-1.871)	1.597 (1.480-1.725)	1.635 (1.515-1.767)
Meal	6165/606	1.596 (1.490-1.711)	1.721 (1.603-1.849)	1.495 (1.391-1.609)	1.624 (1.508-1.750)
Housework	6165/878	1.548 (1.452-1.651)	1.588 (1.489-1.696)	1.463 (1.368-1.566)	1.507 (1.409-1.613)
Other					
Jogging 1km	6165/3742	1.721 (1.607-1.846)	1.873 (1.745-2.013)	1.472 (1.372-1.582)	1.637 (1.523-1.763)
Walking 1km	6165/1286	1.792 (1.681-1.913)	1.815 (1.702-1.938)	1.586 (1.484-1.697)	1.627 (1.522-1.740)
Walking 100m	6165/484	1.738 (1.615-1.873)	1.619 (1.506-1.743)	1.589 (1.472-1.718)	1.474 (1.366-1.591)
Chair	6165/1988	1.375 (1.301-1.455)	1.593 (1.502-1.691)	1.289 (1.217-1.367)	1.520 (1.430-1.619)
Climbing	6165/2887	1.495 (1.410-1.588)	1.693 (1.591-1.803)	1.334 (1.255-1.419)	1.541 (1.446-1.645)
Stooping	6165/2299	1.470 (1.389-1.558)	1.706 (1.605-1.815)	1.340 (1.264-1.424)	1.585 (1.489-1.690)
Lifting 5kg	6165/1013	1.612 (1.514-1.719)	1.671 (1.568-1.784)	1.393 (1.303-1.489)	1.468 (1.373-1.571)
Picking	6165/332	1.312 (1.208-1.421)	1.360 (1.254-1.472)	1.260 (1.152-1.372)	1.314 (1.206-1.429)
Arm	6165/770	1.375 (1.290-1.466)	1.430 (1.341-1.526)	1.324 (1.237-1.417)	1.383 (1.293-1.481)

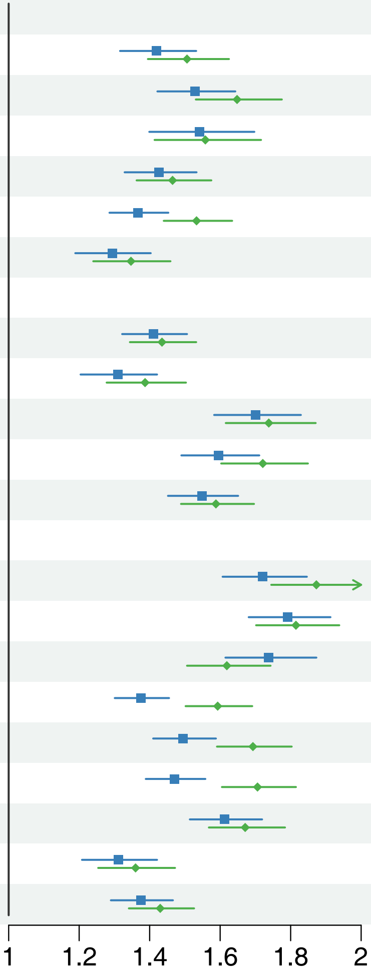

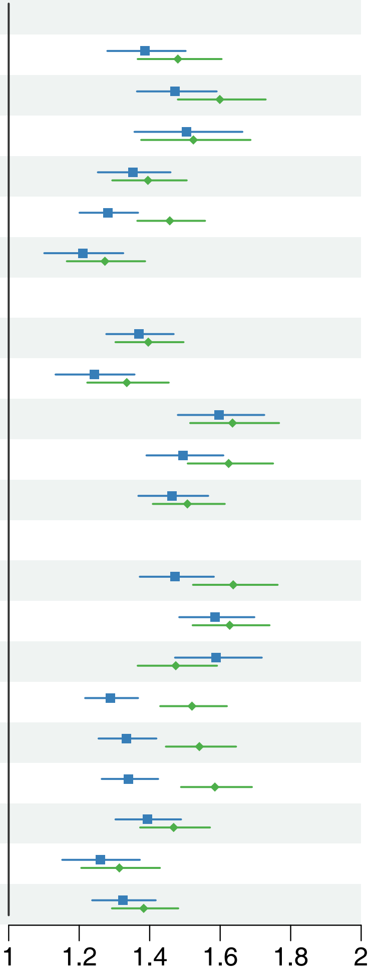

Figure S6

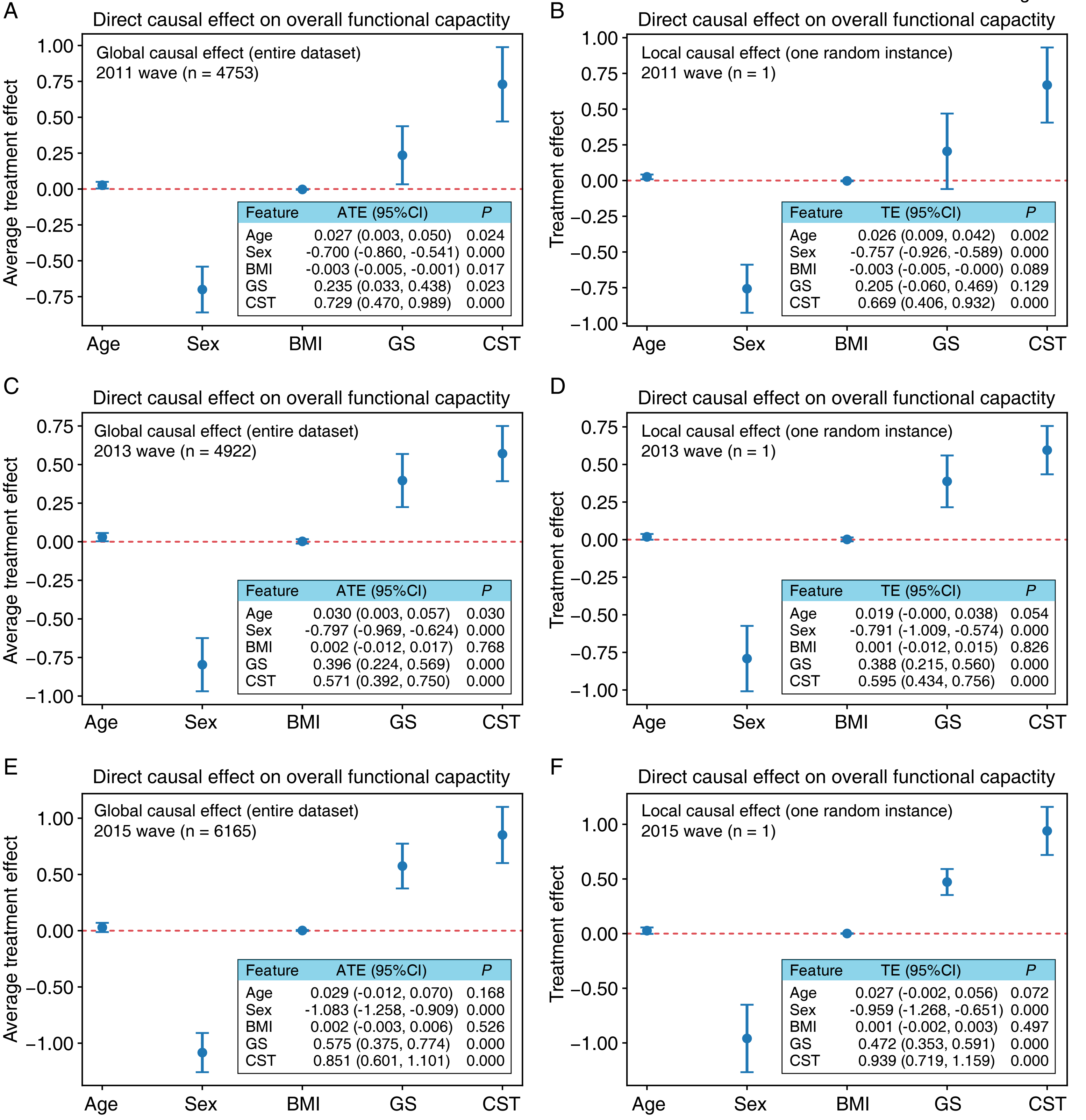
A
Direct causal effect on overall functional capactity
Global causal effect (entire dataset)
2011 wave (n = 4753)
Average treatment effect
1.00
0.75
0.50
0.25
0.00
−0.25
−0.50
−0.75
Feature ATE (95%CI) P
Age 0.027 (0.003, 0.050) 0.024
Sex -0.700 (-0.860, -0.541) 0.000
BMI -0.003 (-0.005, -0.001) 0.017
GS 0.235 (0.033, 0.438) 0.023
CST 0.729 (0.470, 0.989) 0.000
Age
Sex
BMI
GS
CST
B
Direct causal effect on overall functional capactity
Local causal effect (one random instance)
2011 wave (n = 1)
Treatment effect
1.00
0.75
0.50
0.25
0.00
−0.25
−0.50
−0.75
−1.00
Feature TE (95%CI) P
Age 0.026 (0.009, 0.042) 0.002
Sex -0.757 (-0.926, -0.589) 0.000
BMI -0.003 (-0.005, -0.000) 0.089
GS 0.205 (-0.060, 0.469) 0.129
CST 0.669 (0.406, 0.932) 0.000
Age
Sex
BMI
GS
CST
C
Direct causal effect on overall functional capactity
Global causal effect (entire dataset)
2013 wave (n = 4922)
Average treatment effect
0.75
0.50
0.25
0.00
−0.25
−0.50
−0.75
−1.00
Feature ATE (95%CI) P
Age 0.030 (0.003, 0.057) 0.030
Sex -0.797 (-0.969, -0.624) 0.000
BMI 0.002 (-0.012, 0.017) 0.768
GS 0.396 (0.224, 0.569) 0.000
CST 0.571 (0.392, 0.750) 0.000
Age
Sex
BMI
GS
CST
D
Direct causal effect on overall functional capactity
Local causal effect (one random instance)
2013 wave (n = 1)
Treatment effect
0.75
0.50
0.25
0.00
−0.25
−0.50
−0.75
−1.00
Feature TE (95%CI) P
Age 0.019 (-0.000, 0.038) 0.054
Sex -0.791 (-1.009, -0.574) 0.000
BMI 0.001 (-0.012, 0.015) 0.826
GS 0.388 (0.215, 0.560) 0.000
CST 0.595 (0.434, 0.756) 0.000
Age
Sex
BMI
GS
CST
E
Direct causal effect on overall functional capactity
Global causal effect (entire dataset)
2015 wave (n = 6165)
Average treatment effect
1.00
0.50
0.00
−0.50
−1.00
Feature ATE (95%CI) P
Age 0.029 (-0.012, 0.070) 0.168
Sex -1.083 (-1.258, -0.909) 0.000
BMI 0.002 (-0.003, 0.006) 0.526
GS 0.575 (0.375, 0.774) 0.000
CST 0.851 (0.601, 1.101) 0.000
Age
Sex
BMI
GS
CST
F
Direct causal effect on overall functional capactity
Local causal effect (one random instance)
2015 wave (n = 1)
Treatment effect
1.00
0.50
0.00
−0.50
−1.00
Feature TE (95%CI) P
Age 0.027 (-0.002, 0.056) 0.072
Sex -0.959 (-1.268, -0.651) 0.000
BMI 0.001 (-0.002, 0.003) 0.497
GS 0.472 (0.353, 0.591) 0.000
CST 0.939 (0.719, 1.159) 0.000
Age
Sex
BMI
GS
CST
